# VMamba-QAG-net: a five-stage pipeline for SYNTAX score computation and decision support in interventional cardiology using X-ray angiography

**DOI:** 10.3389/frai.2026.1903775

**Published:** 2026-09-23

**Authors:** G. Sunilkumar, P. Kumaresan

**Affiliations:** 1 School of Computer Science Engineering and Information Systems (SCORE) Vellore Institute of Technology, Vellore, India; 2 Centre For Artificial Intelligence Research (C-FAIR) Vellore Institute of Technology, Vellore, India

**Keywords:** coronary artery disease, EfficientNetV2S, EfficientSS2D, VMamba-QAG-net, X-ray coronary angiography image (XCA)

## Abstract

Coronary Artery Disease (CAD) remains a significant worldwide health problem, making accurate evaluation of disease severity essential for treatment planning. The SYNTAX score, derived from X-ray coronary angiography (XCA) images, plays a key role in guiding treatment decisions. However, manual scoring is time-consuming, subject and prone to inter-observer variability, necessitating the development of an objective and reliable system. We proposed VMamba-QAG-Net, a novel five-stage pipeline designed for SYNTAX score estimation directly from XCA images. Stage 1 performs binary vessel segmentation to resolve extreme class imbalance, using a preprocessing of Bilateral Filtering, CLAHE, and Unsharp Masking to enhance vessel boundaries, an EfficientSS2D block with parallel five direction visual mamba scanning and Quantum Attention Gate (QAG) to preserve long-range vascular connectivity. Stage 2, executes multi-class anatomical labelling into 27 distinct coronary artery segments using a four-channel guided input, supported by a Vessel Prototype Memory Bank with metric learning and a centreline-weighted skeleton loss for thin distal structures. Stage 3 and 4 extract ordered vessel centrelines via skeletonization, calculates percentage diameter stenosis through perpendicular ray casting, and detects grade stenotic lesions, using Aquila Optimiser. Stage 5 combines the stenosis information to calculate the SYNTAX score and support treatment decisions in revascularisation. Our proposed model was evaluated on the ARCADE dataset and attained a Dice coefficient of 0.9070 and accuracy of 0.9841 for binary segmentation. For 27-class coronary artery segmentation achieved a Dice coefficient of 0.7512, accuracy of 0.9514, and present class Dice of 0.8214. These results demonstrate the effectiveness of the proposed framework and its potential to support cardiologists in clinical decision-making.

## 1 Introduction

Coronary Artery Disease (CAD) continues to be one of the most serious health burdens worldwide, claiming nearly nine million humans are losing their lives from this risk every year and placing significant strain on healthcare systems in both developed and developing countries. As a leading form of cardiovascular disease (CVD), CAD contributes substantially to a wider CVD burden, which caused an estimated 19.8 million deaths in 2022, approximately 32% of all deaths worldwide (World Health Organisation, 2024). CAD alone accounted for 164.0 million DALYs in 2015 (Ralapanawa and Sivakanesan, 2021), and affected an estimated 315 million people globally in 2022 (Stark et al., 2024). The primary cause of CAD is atherosclerosis, a condition in which calcium, fat and fibrous tissue gradually accumulate along the inner walls of coronary arteries, forming deposits known as plaques. Over time, these plaques narrow the coronary arteries and reduce blood flow to the heart, a condition clinically referred to as stenosis. When the heart muscle does not receive enough oxygen-rich blood, and patients may experience angina, shortness of breath, and other cardiac symptoms (Pandey et al., 2020). Although genetics plays an important role in CAD, the disease is mostly caused by lifestyle-related risk factors such as high blood pressure, age, gender, elevated heart rate, diabetes, and unhealthy daily habits. Since CAD often does not show clear symptoms in the early stages, early identification and better medical treatment are important to improving patient outcomes and survival rates.

X-ray coronary angiography (XCA) is the gold-standard cardiac imaging approach used to diagnose CAD and guide therapy recommendations such as percutaneous coronary intervention (PCI), a widely performed clinical procedure used to widen the stenotic arteries and improve circulation to the heart. During these procedures, cardiologists utilize real-time XCA to locate and measure blockages in the coronary arteries. However, angiographic percent-stenosis is an unreliable predictor of whether a blockage is actually restricting blood flow, particularly in the intermediate range of roughly 40 to 70% blockage. For these intermediate lesions, cardiologists confirm functional significance using Fractional Flow Reserve (FFR) a pressure based physiological test with a standard intervention threshold of ≤ 0.80 (Mahmoudi et al., 2025). The clinical importance of FFR is well established. Pijls et al. (1996) demonstrated that FFR can accurately identify blockages that significantly reduce blood flow and showed good agreement with standard non-invasive cardiac tests. The FAME trail found that FFR-guided sent placement provided better outcomes than angiography alone in patients with multiple coronary blockages (Tonino et al., 2009). Similarly, the FAME2 trail showed that FFR-guided stenting combined with medical therapy was more effective than medical therapy alone in patients with stable CAD and flow-limiting blockages (De Bruyne et al., 2014). After confirming whether a blockage is functionally significant, the treatment choice depends on the complexity of the coronary lesions. The SYNTAX trail demonstrated that CABG resulted in fewer major cardiac complications than PCI in patients with complex CAD (Serruys et al., 2009). These findings are incorporated into the 2021 ACC/AHA/SCAI guideline for Coronary artery revascularization, which provides guidance for selecting between PCI and CABG (Writing Committee Members et al., 2022). However, reading these images accurately is difficult and often subjective. Since the heart is constantly beating and blood vessels frequently overlap, even experts can find it difficult to get a clear and consistent view of the damage. Artificial Intelligence helps by creating detailed images of the heart’s blood vessels, allowing doctors to identify CAD quickly and accurately.

Machine Learning (ML) models have shown good results in detecting CAD, but their reliability can be affected by limited datasets, class imbalance, risk of overfitting, and poor performance in diverse patient population, particularly in complex clinical cases such as patients with pacemakers (Rehman et al., 2025). Recent advances in deep learning (DL) have led to various approaches for coronary artery segmentation and stenosis detection using XCA images. Gao et al. (2022) proposed an ensemble model combining DL and filter-based features to detect stenosis in XCA images, while Serrano-Antón et al. (2025) applied clustering and graph theory techniques to segment the coronary arteries. Kaba et al. (2023) aimed to automate coronary artery segmentation and stenosis classification by using U-Net, ResU-Net-a, UNet++ for the segmentation task, along with DenseNet201, EfficientNet-B0, MobileNetv2, ResNet101, and Xception models for the classification task. Hatfaludi et al. (2024) developed DL model to predict FFR values using non-invasive imaging data from optical coherence tomography and XCA, which reduces the need of invasive diagnostic procedures. Zhang et al. (2026) developed HR-UMamba++ model used to segment coronary arteries in XCA images. Zhao et al. (2025) proposed multi-stream attention- guided coronary analysis network (MACAN) framework with multi-scale channel and spatial attention mechanisms for accurate artery localisation and segmentation.

Recent studies have explored advanced optimisation techniques for medical image analysis. Abualigah et al. (2023) combined the reptile search algorithm with the slap swarm algorithm applied for multi-level thresholds for image segmentation. Pascual-González et al. (2025) showed the use of optuna and Ultralytics for automated hyperparameter tuning. Ismail et al. (2023) used Multi Objective Grass Hopper Optimisation Algorithm (MOGOA) for Alzheimer’s disease classification, while Diab et al. (2025) and Balaha et al. (2025) applied Greylag Goose (GGO) and Archimedes Optimisation Algorithm (AOA) for medical image classification and segmentation tasks. However, many of these approaches suffer from high computational cost, convergence and limited validation on diverse real-world clinical datasets.

Several works have focused on coronary artery centreline extraction and stenosis grading. Epifanov et al. (2025) proposed an end-to-end multitask network for direct vessel centrelines extraction, Wu et al. (2023) and Zhang et al. (2022) developed CNN and C-UNet based approaches for continuous centreline tracking from coronary angiographic images. Mao et al. (2025) utilised dual output CNN-based model to automatically for stenosis severity grading. Padariya et al. (2025) combined UNet3 + with image processing techniques for stenosis detection and measurement. Although these methods attained good performance, most research focus on individual tasks such as segmentation, centreline extraction, or stenosis grading separately, resulting in fragmented clinical workflows. In addition, many existing attention-based methods suffer from signal dilution in thin distal vessels, poor robustness against imaging artefacts and limited capability for automated clinical reporting. Limitations of existing segmentation methods as shown in Table 1.

**Table 1:** Limitations of existing coronary artery segmentation methods on ARCADE dataset.

Work	Method	Limitations
Yang et al. (2026)	UNet	Due to limited dataset performance is affected. It is mainly designed for X-rays and does not perform well on other modality images.
Lalinia et al. (2026)	Swin UNETR (Fine-tuning with label smoothing)	Performance may drop under poor image quality due to noise, low contrast
	Swin UNETR (Adapter-tuning with label smoothing)	
Zhao et al. (2025)	MACAN	Performance drops on challenging validation data. Limited improvement from tuning/ preprocessing. Struggles with complex vessel labelling.
Chen et al. (2025)	SFAGDeepLabv3+	This work enhanced feature extraction and segmentation of complex coronary vessels. Information loss during encoder and decoder transitions leads to discontinuous vessel segmentation.
Ferrari et al. (2025)	CoroSAM Bottleneck 25%	The model’s reliability is limited by the narrow range of training data.

Gap analysis:

1. Existing models such as UNet and CoroSAM are trained using limited datasets with fixed loss weights and fixed augmentation methods, which causes overfitting and low performance on new patient data.
2. Standard attention—based networks and cross-entropy classifiers focus more on large vessel areas, causing poor segmentation or missing of thin and important distal vessel segments.
3. Transformer based models do not perform proper image enhancement before feature extraction, leading to incorrect vessel boundaries and broken vessel tracking in low-contrast XCA images.
4. Existing 4-direction recurrent and attention networks process features sequentially, which reduces GPU parallelization and missing diagonal vessel information, and breaks continuous vessel connectivity due to rigid gating mechanisms.
5. Multi-class cross-entropy models often confuse nearby or overlapping vessel territories because they do not include anatomical structural constraints.
6. Most of the existing methods only generate segmentation masks and do not provide integrated pipelines for centreline extractions, stenosis analysis, and automated clinical reporting.

To address these limitations, this work proposed VMamba-QAG-Net, a unified five-stage framework that combines vessel detection, 27-class anatomical segmentation, centreline extraction, multi-grade stenosis classification, and automated SYNTAX score generation. Unlike existing fragmented approaches, the proposed framework introduces an SS2D Block based on Visual Mamba with parallel 5-direction scanning to preserve long range spatial continuity and improve thin vessel representation across complex coronary structures. Furthermore, a Quantum Attention Gate (QAG) is designed to eliminate rigid gating mechanisms and maintain continuous vessel connectivity during decoder feature fusion.

The major contributions of this research work are as follows:

1. A preprocessing pipeline using Bilateral Filtering, CLAHE, and Unsharp Masking is introduced to enhance low-contrast vessel boundaries, while Attention- Guided Dropout (AGDrop) preserves important coronary vessel features and suppresses noise in background
2. We proposed an EfficientSS2D Block based on Visual Mamba with parallel 5-direction scanning to capture horizontal, vertical and diagonal vessel information efficiently, paired with a Quantum Attention Gate (QAG) that preserves continuous vessel connectivity during decoder feature fusion.
3. A Swarm-Based Hyperparameter Optimiser (SBHO) is introduced to automatically optimise augmentation settings and loss weights, while test-time augmentation (TTA) improves robustness and reduces overfitting on unseen patient data.
4. A vessel prototype memory bank with metric learning and centreline-weighted skeleton loss is proposed to improve learning of rare and thin distal vessel segments such as D2a and OM2a by strengthening feature representation and enhancing vessel centreline gradients.
5. Three multi-scale auxiliary decoder heads are proposed to classify five coarse vascular territories, improving anatomical boundary learning and reducing cross- territory vessel misclassification.
6. A unified five-stage pipeline is introduced to integrate vessel segmentation, 27-class coronary artery labelling, centreline extraction, stenosis severity grading, SYNTAX score calculation, and revascularisation treatment recommendation.
7. A comprehensive evaluation is performed using Dice Coefficient, Jaccard Index, Accuracy, Precision, Recall, Specificity for segmentation performance, with stenosis severity estimation, and SYNTAX score computation reported as characterised pipeline outputs.
8. We proposed present-class Dice metric that evaluates coronary artery segmentation by considering only the vessels are present in the image.

## 2 Related work

Almansour et al. (2025) used swarm -based optimisation is utilised to tune CNN hyperparameters such as learning rate and batch size to improve the performance. Aguerchi et al. (2024) used swarm optimisation to find the best CNN architecture and hyperparameters for accurate breast cancer image classification. Saifullah et al. (2026) used to adjust learning rate, dropout, and U-Net parameters to improve segmentation accuracy and reduce overfitting. Ma et al. (2025) and Nazzal et al. (2024) showed that Test-Time Augmentation (TTA) enhances segmentation accuracy and robustness by using multiple augmented test image variations during inference.

Ismael et al. (2025) showed that CLAHE improves contrast in low-contrast medical images while preserving anatomical details. Rizwana et al. (2025) and Raja and Ramadevi (2022) demonstrated that unsharp masking improves edge details, sharpness, and lesion visibility. Zhang et al. (2023), Li et al. (2023), and Taassori (2024) showed that Bilateral Filtering reduces noise while preserving edges, tissue structures, and important anatomical details.

Zhang et al. (2024) proposed Visual Mamba based State Space Models (SSMs) for efficient long-range spatial dependency learning with linear computational complexity. Xu et al. (2025) proposed efficient Mamba (EMB), which enhances Vision Mamba using TransSSM and Window Spatial Attention (WSA) for enhanced local and global feature extraction with fewer parameters and FLOPs. Sun et al. (2025) developed Dual-encoder Global–Local Feature Extraction Network (DGFE)-Mamba, combining CNN-based local feature extraction with Mamba-based global dependency modelling for accurate medical image segmentation. Ren et al. (2026) introduced Contextual and Frequency-Guided Mamba Network (CFG-MambaNet), which uses Mamba -based state space blocks and frequency-guided adaptive context modules to improve long-range dependency learning feature representation and boundary segmentation in medical images.

Recent works have shown that quantum inspired DL improves medical image analysis enhancing feature learning, segmentation, and image generation. Pandey and Mandal (2025) used Quantum Attention Mechanism (QAM) to improve feature selection and classification accuracy. Wang and Lim (2025) proposed Quantum Tensor Entanglement Attention (Q-TEA) and Quantum residual connections (QR) to improve spatial-channel feature interaction and preserve boundary continuity in low-contrast and noisy images. Khawaji et al. (2025) proposed Quantum Transformer Learning (QTL) to improve high-resolution medical image generation while reducing augmentation artifacts and preserving fine image details for computer-aided diagnosis (CAD).

The remainder of this paper is organized as follows. Section 2 reviews the related work in coronary artery segmentation. Section 3 presents the methodology, Binary vessel segmentation, 27-class vessel multi segmentation. Post processing pipeline, stenosis grading and SYNTAX score estimation. Section 4 describes the experimental settings, dataset description, implementation details, evaluation metrics. Section 5 discusses the experimental results, compares the SOTA models, ablation studies, highlights key findings. Section 6 details the discussion of the entire work and Section 7 concludes the paper by summarizing the main contributions and findings.

## 3 Methodology

The proposed VMamba-QAG-Net is five stage Deep Learning (DL) framework as shown in the Figure 1 designed for coronary artery segmentation and SYNTAX score estimation from XCA images using the ARCADE dataset. In Stage 1, binary vessel segmentation is performed to generate a soft vessel probability map, which is concatenated with the original XCA image to form a 4-channel guided input for Stage 2. Stage 2 performs 27-class multi vessel segmentation, where each vessel pixel is assigned to one of the 26 coronary artery segments defined in the ARCADE SYNTAX protocol, while the remaining class represents as background. Stages 3 to 5 further carry out vessel centreline extraction and stenosis detection, vessel-diameter based stenosis severity estimation, and final SYNTAX score calculation. The complete work flow of the proposed pipeline is shown in the Figure 1.

**Figure 1:**
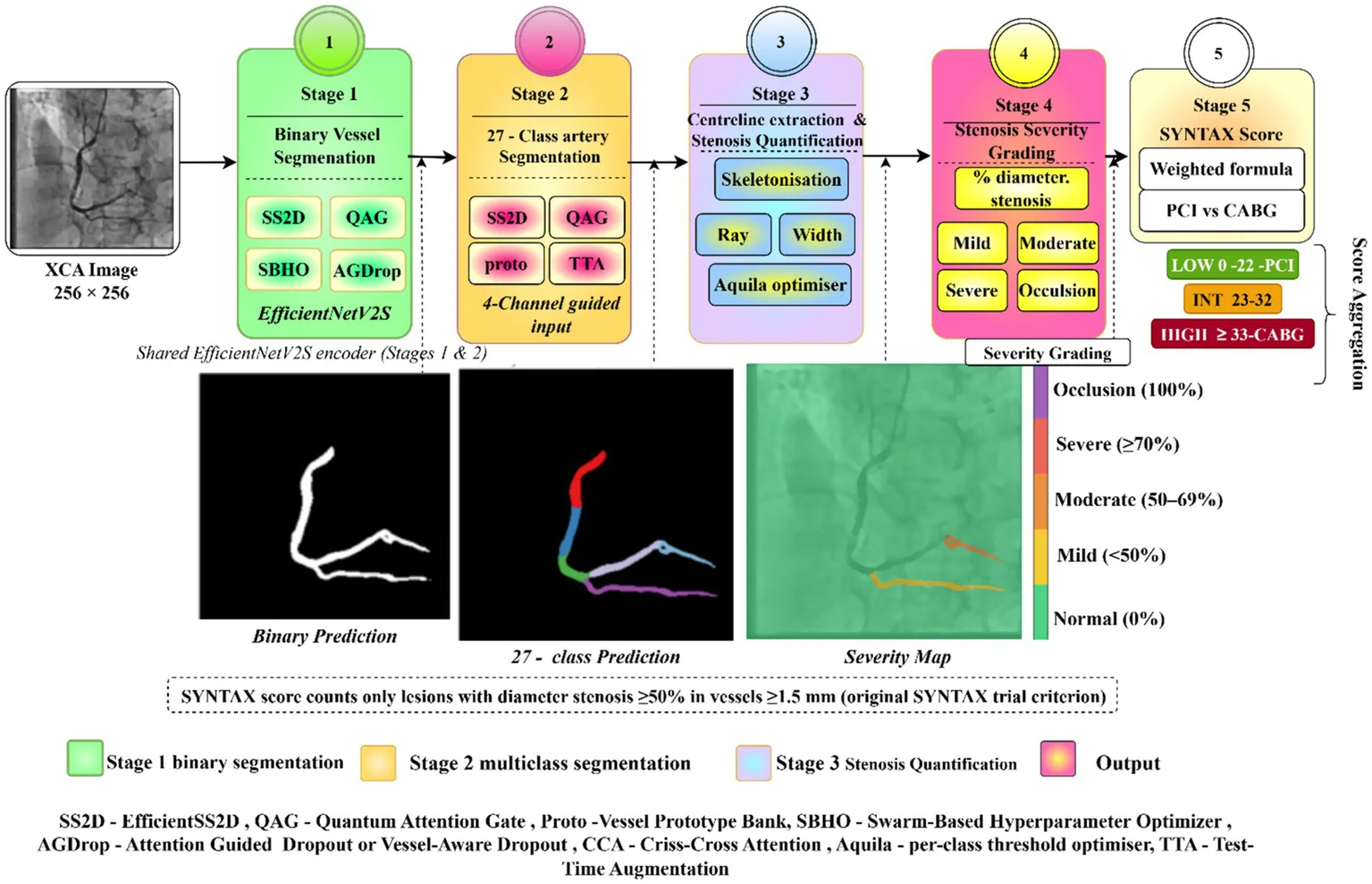
The proposed VMamba-QAG-Net architecture for coronary analysis.

### 3.1 Stage 1: binary vessel segmentation

Stage 1 as shown in the Figure 2 identifies the presence and absence of coronary vessels at the pixel level of the input angiographic image. Given a pre-processed XCA image $X\in R^{H\times W\times 3}$ , the model produces a binary probability map $P_{1}\in {\left[0,1\right]}^{H\times W}$ indicating foreground coronary vessel pixels. This probability map is subsequently used as a fourth guidance channel in Stage 2.

**Figure 2:**
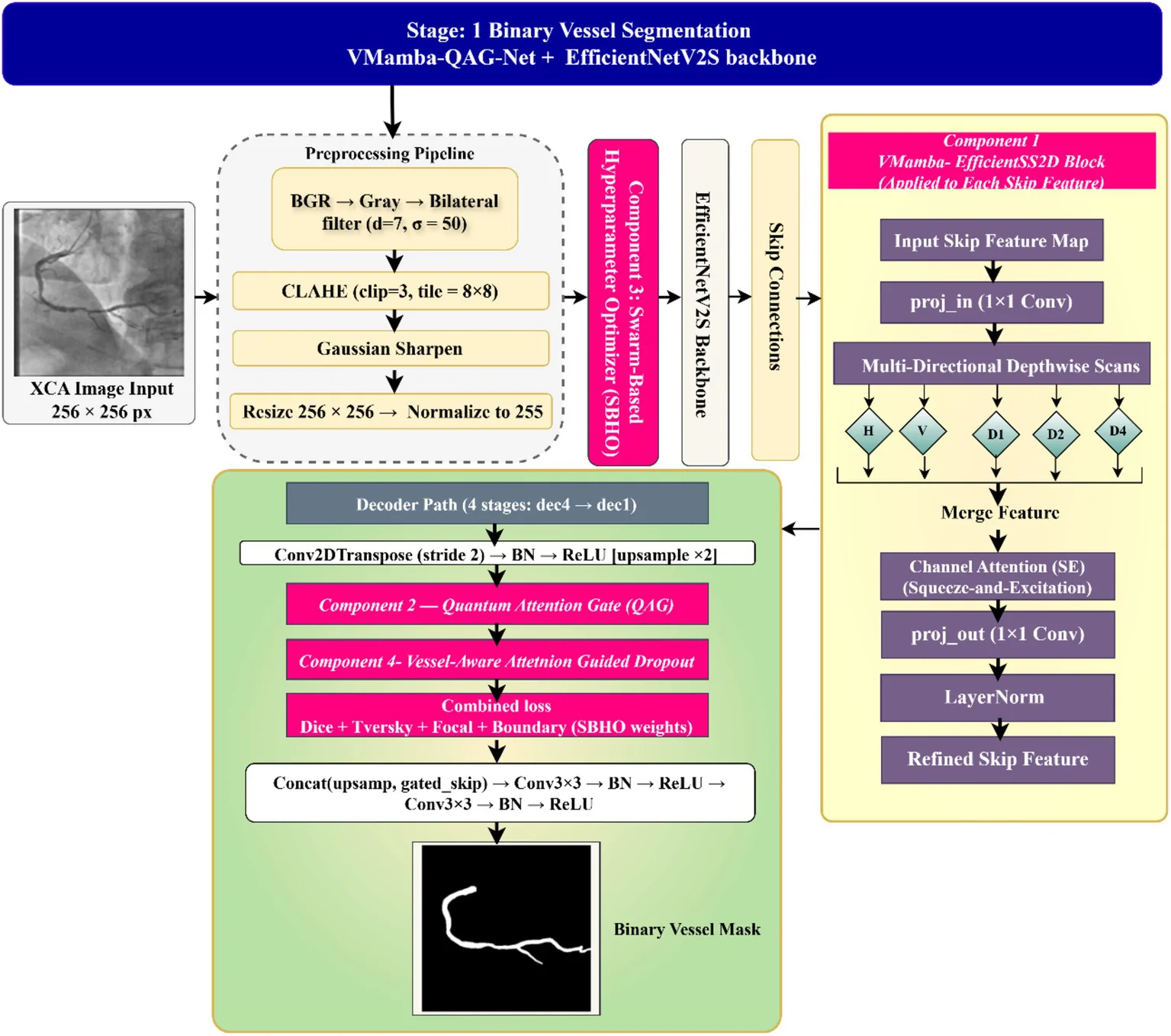
Overview of the proposed stage 1 binary vessel segmentation workflow.

#### 3.1.1 XCA image preprocessing

All input images were passed through a fixed preprocessing pipeline before being fed into the network. First, a bilateral filter of d = 7, $\sigma_{\text{color}}=50,\sigma_{\text{space}}=50$ was applied to the XCA grayscale inputs to suppress noise while preserving vessel edges. Next, contrast-limited adaptive histogram equalization (CLAHE) with a clip limit of 3.0 and an 8 × 8 tile grid was utilised to enhance local contrast in low-intensity coronary vessel regions. After that, Unsharp masking was then applied to make thin vessel boundaries sharper. Finally, each image was converted to RGB format, resized to 256 × 256 pixels were and normalized to the range [0,1].

#### 3.1.2 EfficientNetV2-S encoder

The encoder utilizes an ImageNet pretrained EfficientNetV2-S backbone. Four intermediate feature maps are extracted as skip connections at progressively reduced spatial resolutions of 64 × 64(64 channels), 32 × 32 (96 channels), 16 × 16 (128 channels), 8 × 8 (192 channels). These feature maps capture multi-scale vascular representations across varying network depths. Training was conducted in a two-phase strategy. In phase 1, backbone weights are frozen to stabilize decoder optimisation and prevent gradient disruption to the pretrained features. In phase 2, the overall network was fine-tuned end-to-end to jointly optimise the encoder and decoder representations.

#### 3.1.3 Component: EfficientSS2D block

Each skip connection is processed through the proposed EfficientSS2D block prior to entering the decoder. Unlike traditional convolutional blocks that use isotropic receptive fields, EfficientSS2D captures long-range spatial relationships along important vessel directions using five directional convolution branches. Here, SS2D refers to spatial scanning through convolutions rather than recurrent state-space models.

Let $X\in R^{H\times W\times C_{\text{in}}}$ represent an encoder feature map. First, a 1 × 1 convolution is applied to project X into C channels. Five parallel depthwise convolution (DWConv) branches subsequently operate on these projected features, whose branch-wise transformations defined in Equation 1–5.

$$h=\text{DWCon}v_{1\times 15}\;(X_{\text{proj}})$$ (1)

$$v=\text{DWCon}v_{15\times 1}\;(X_{\text{proj}})$$ (2)

$$\;d_{1}=\text{DWCon}v_{3\times 3,r=1}\;(X_{\text{proj}})$$ (3)

$$d_{2}=\text{DWCon}v_{3\times 3,r=2}\;(X_{\text{proj}})$$ (4)

$$d_{4}=\text{DWCon}v_{3\times 3,r=4}\;(X_{\text{proj}})$$ (5)

Here, *r* is the dilation rate. The horizontal (h) and vertical (v) branches capture elongated coronary vessel patterns, while the dilated branches d_1_, d_2_, d_4_ enlarge the receptive field to 3 × 3, 5 × 5, and 9 × 9 pixels, modelling multi-scale vessel junctions and bifurcations. The horizontal and vertical branches use elongated strip kernels (1 × 15 and 15 × 1) instead of square kernels. Since coronary vessels are thin and linear, these kernels capture vessel information along the vessel direction while suppressing the background noise. The horizontal branch (1 × 15) responds to near-horizontal vessel runs and the vertical branch (15 × 1) to near-vertical runs. The long-range behaviour of the H and V branches arises from the elongated shape of the strip kernels rather than from a large square receptive field, which helps preserve vessel continuity even in low-contrast regions. At bifurcations, vessels branch in different directions, making a single directional kernel less effective. The dilated 3 × 3 kernels (r = 1,2,4) instead provide multi-scale, wider context to capture vessel junctions. Each branch uses Batch Norm and ReLU activation, and their outputs are combined by element-wise summation followed by a feature fusion layer, as defined in Equation 6.

$$M=\mathrm{Con}v_{\text{merge}}\;(h+v+d_{1}+d_{2}+d_{4})$$ (6)

To optimise inter channel dependencies, a channel attention mechanism recalibrates M through global average pooling (GAP) followed by multilayer perceptron (MLP) bottleneck two convolutions (*C*/8 channels with ReLU; *C* channels with sigmoid), producing a channel weight vector $w\in {\left[0,1\right]}^{C}$ , as defined in Equations 7, 8.

$$w=\sigma (W_{2}\;(\text{ReLU}(W_{1}(\mathrm{GAP}\;(M)))))\;$$ (7)

$${'}^{M}=M⊙w$$ (8)

Finally, the modulated feature map *Ḿ* is projected back to $C_{\text{in}}$ Channels through a 1 × 1 convolution, added to the residual input *X*, and normalized with layer normalization to ensure stable gradient flow, as defined in Equation 9.

$$Y=\mathrm{LN}(\mathrm{Con}v_{\text{out}},({'}^{M})+X)$$ (9)

The architectural design preserves fine-grained vascular topologies while ensuring stable gradient propagation during training.

The proposed EfficientSS2D blocks Figure 3 is integrated across all four resolution scales (k 𝜖 {1,2,3,4}) of the decoder network to balance performance with computational speed. At each level, the block processes the incoming low-level skip features $X_{k}$ before they merge with the upsampled deeper features. This systematic filtering corrects spatial alignment errors and removes background noise right before the decoder reconstructs the final image. To prevent high computational overhead, the block replaces standard 2D convolutions with depthwise separable operations.

**Figure 3:**
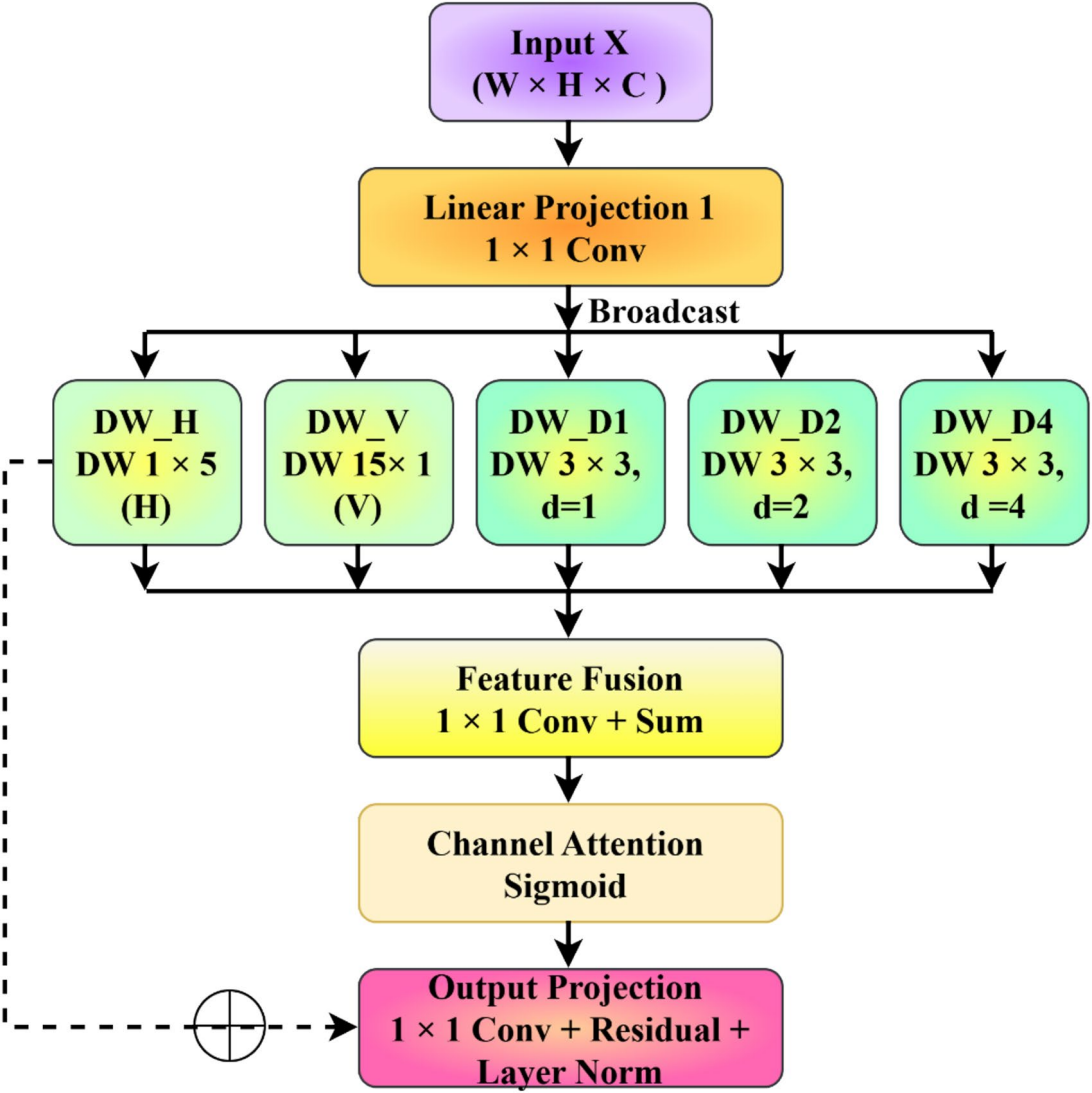
Architectural diagram of the proposed EfficientSS2D block.

Standard Convolution Complexity: For a kernel of size $K_{H}\times K_{H}$ , a traditional dense layer requires a parameter cost proportional to $O(K_{H}.K_{w}.C^{2}$ ).

Depthwise Convolution Complexity: The depthwise alternative drops this cost to $O(K_{H}.K_{w}.C$ ), completely removing the squared channel factor $C^{2}$ .

A multi-scale spatial core processes these features using five parallel depthwise convolution branches horizontal (DW 1 × 15), vertical (DW 15 × 1), and three dilated variants (DW 3 × 3 with d = 1,2,4) to isolate multi-directional coronary vessel tracks and bifurcations. Branch outputs are integrated through element-wise summation followed by a 1 × 1 feature fusion layer. A squeeze-and-excitation channel attention module GAP which is subsequently mapped through MLP bottleneck to reduce the channel dimensionality to C/8 before scaling back to C channels through sigmoid activation function dynamically recalibrates inter- channel responses before the final feature map is projected back to $C_{\text{in}}$ channels, integrated with the identity skip path through a residual connection, and stabilized with layer normalisation.

Table 2 reports the branch ablation of the EfficientSS2D block. The full configuration attains the best performance across all metrics, confirming that all five branches contribute. Removing either branch group reduces performance: The strips-only variant (not dilated) drops to 0.7512 Dice and the dilated-only variant (no strips) to 0.7445 Dice, showing that the directional and multi-scale branches are complementary rather than redundant. The dilated-only configuration also produces the largest clDice reduction (0.7682 versus 0.8120), indicating that the directional strip branches contribute most to vessel connectivity. Removing a single strip (No H or No V) causes a smaller but consistent decrease, confirming that both directional branches carry useful information.

**Table 2:** Ablation of EfficientSS2D branch configurations (stage-1, ARCADE test set).

Model variant	SS2D branches	Dice	IoU	Accuracy	Precision	Recall	Specificity	clDice
Full EfficientSS2D	H + V + D1 + D2 + D4	0.7886	0.6651	0.9536	0.7591	0.8051	0.9624	0.8120
No dilated (strips only)	H + V	0.7512	0.6120	0.9450	0.7240	0.7745	0.9552	0.7750
No strips (dilated only)	D1 + D2 + D4	0.7445	0.6015	0.9432	0.7180	0.7680	0.9538	0.7682
No H	V + D1 + D2 + D4	0.7620	0.6301	0.9485	0.7350	0.7862	0.9580	0.7850
No V	H + D1 + D2 + D4	0.7654	0.6342	0.9492	0.7382	0.7895	0.9589	0.7884

#### 3.1.4 Component: quantum attention gate (QAG)

Standard attention gates in medical image segmentation use spatial weights to reduce irrelevant features in skip connections. The proposed QAG improves this by adding a quantum- inspired phase modulation, allowing smoother and more flexible attention instead of simple amplitude-based selection. Let $X\in R^{H\times W\times C_{x}}$ denote the encoder skip feature map and $G\in R^{\dot{H}\times \dot{W}\times C_{g}}$ the decoder gating signal. If $H\neq \dot{H}$ . Features are processed sequentially across two branches.

- Amplitude Path: Input features are projected via 1 × 1 conv to C/2 channels and aggregated into a spatial weight map αϵ[0,1] , as calculated in Equation 10

$$\alpha =\mathrm{AMP}\_\mathrm{AGG}((W_{x,\mathrm{amp}}(x)+W_{g,\mathrm{amp}}(g)))$$ (10)

Where AMP_AGG applies a 1 × 1 conv, batch normalisation, and a ReLU activation.

- Phase Path: To condition phase shifts on amplitude features, the aggregated space is projected to C/2 channels to calculate the localized phase map φ, is calculated in Equation 11.

$$\varphi =\mathrm{PHS}\_\mathrm{AGG}((W_{x,\mathrm{phs}}(X)+W_{g,\mathrm{phs}}(G))).\psi$$ (11)

Where, PHS_AGG applies a 1 × 1 convolution, Batch Normalisation, and a ReLU activation. The parameter phase scale (𝜓) is a trainable scalar initialized to ᴨ.

The final gate uses a cosine pattern that behaves as quantum wave interference are calculated by using Equations 12, 13.

gate=ReLU(α.cos(φ.Π)) (12)

$$x_{\text{out}}=x⊙\text{gate}$$ (13)

When the phase φ.Π=0 , it triggers constructive interference (cos (0) = 1), allowing full amplitude pass- through. When φ.Π=Π/2 , it triggers destructive interference (cos(ᴨ/2) = 0), completely reducing background noise regardless of amplitude.

As shown in Figure 4, the QAG pipeline takes two inputs: the encoder skip feature (x) the decoder gating signal (g). First, in the amplitude branch, both features pass through 1 × 1 convolutions and are combined in the AMP_AGG block to create spatial weights α. This outcome is then pass to the phase branch, where the features are processed again through another set of convolutions and the PHS_AGG block to generate a phase map. The phase map is scaled using a trainable parameter initialized to ᴨ. Finally, the amplitude and phase maps are merged using a cosine-based modulation function to produce the gated output feature map x_out with a residual shortcut connection.

**Figure 4:**
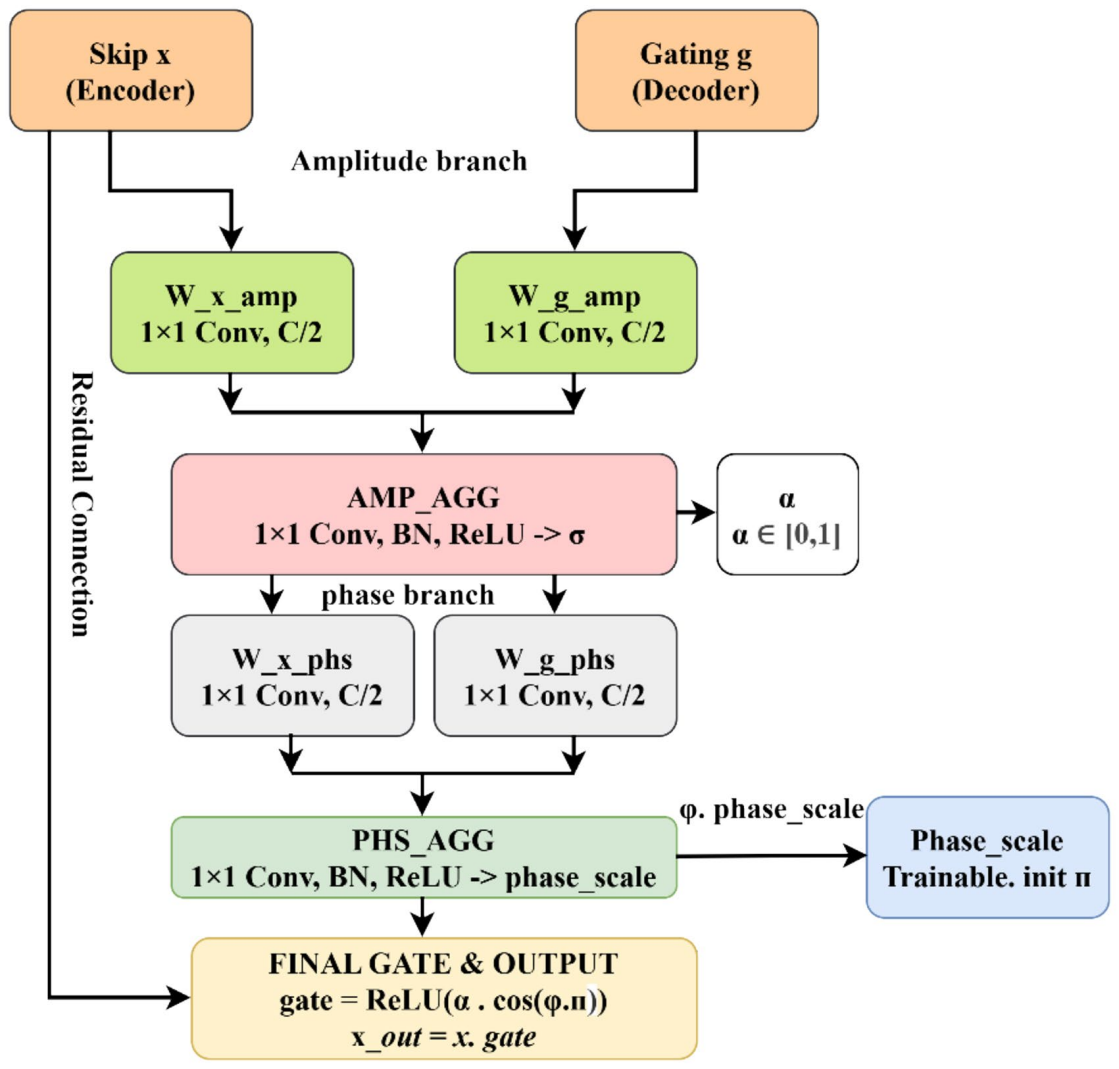
Schematic architecture of the quantum attention gate.

#### 3.1.5 Component: vessel aware attention-guided dropout (AGDrop)

The proposed regularization technique selectively removes spatial activations based on anatomical structure confidence. The process is mathematically defined in Equations 14–17.

$$V_{p}=\sigma \;(\mathrm{Con}v_{3\times 3}(x))\epsilon {\left[0,1\right]}^{H\times W\times 1\;\;}\;$$ (14)

$$P_{\text{keep}}=1-p_{\text{base}.\;}(1-0.8.V_{p}),p_{\text{base}}=0.1$$ (15)

$$M_{B}∼\text{Bernoulli}(P_{\text{keep}}),M_{B}\in {\left\{0,1\right\}}^{H\times W\times 1\;\;}\;$$ (16)

$$X_{\text{drop}}=\frac{X.^{'}M_{B}}{\text{mean}(P_{\text{keep}})+\in }$$ (17)

Where, $M_{B}$ is the spatial binary mask, $M_{B}$ tiled across all C channels, and ε is a small constant for numerical stability during expectation renormalization.

Traditional neural networks often have difficulty segmenting medical images because they remove fine boundaries with hard attention operations or damage delicate structures such as blood vessels through random dropout. To address this, the QAG treats features such as waves and uses phase adjustment with cosine modulation to preserve soft anatomical boundaries more smoothly.

The vessel aware AGDrop component further enhances this by calculating an adaptive survival rate that removes background noise while protecting important vascular structures during training. Finally, the multi-path decoder fusion connects these components together, combining stable amplitude features with phase information and merging multi-scale feature paths to produce an anatomically accurate and stable final output map.

#### 3.1.6 Decoder architecture

The decoder uses a four-level U-Net structure with three skip connections. At each level, the feature maps are upsampled using transposed convolution, while the corresponding encoder skip features are filtered using QAG. The gated features are then refined with AGDrop layer to remove noise while preserving important anatomical features. After that, the up sampled and regularized features are concatenated in channel wise and processed through 3 × 3 conv to batch normalisation and ReLU layers for feature fusion.

#### 3.1.7 Component: swarm-based hyperparameter optimiser (SBHO)

Manually setting loss weights and augmentation parameters for imbalanced vessel segmentation is challenging because different objectives can conflict with each other. To solve this, a swarm-based hyperparameter optimiser automatically searches four parameter spaces before training as also detailed in Table 3. The fitness function used to evaluate each candidate solution is defined in Equation 18.

$$\begin{array}{l} f=w_{t}\cdot 0.40+w_{d}\cdot 0.35+w_{f}\cdot 0.15+w_{b}\cdot 0.10-i\sum \lambda_{i}\\ |w_{i}-w_{i}^{*}|-100\cdot w_{\mathrm{bg}}+\eta_{1}\;e^{-|\mathrm{rot}-20|/10}+\eta_{2}\;e^{-|\beta -0.82|/0.05} \end{array}$$ (18)

**Table 3:** SBHO hyperparameter search spaces.

Space	Parameter	Distribution	Role
1	Loss Weights ( $W_{\text{dice},}W_{\text{tversky},}W_{\text{focal},}W_{\text{boundary}}$ )	Dirichlet (1,1,1,1)	Relative contribution of each loss term
2	$W(bg_{\text{weight}})$	Uniform (0.0001, 0.01)	Back ground pixel class weight
3	Rot_range	Uniform (0°, 30°)	Maximum augmentation rotation angle
4	Tversky_beta	Uniform (0.70, 0.90)	False negative penalty in Tversky loss

Where, $w_{i}^{*}$ are prior knowledge targets $\lambda_{i}$ are penalty coefficients, and $\eta_{1},\eta_{2}$ reward configurations near empirically validated regions.

The best-scoring agent is identified each iteration, and all other agents move toward it through cooperative attraction mechanism toward the current best solution, as described in Equation 19.

$$\theta_{h\;\;}\leftarrow \theta_{h\;\;}+0.3(\theta_{\text{best}\;\;}-\theta_{h\;\;})+\varepsilon ,\varepsilon ∼(0,\sigma^{2})$$ (19)

The final optimal configuration is passed directly to the loss and augmentation pipeline, eliminating manual hyperparameter tuning entirely. SBHO is prior-guided search rather than a cross-validated procedure: candidates are scored analytically against Equation 18 using empirically-derived prior targets, not by training or validating on data. Because scoring is analytic, no portion of the ARCADE dataset is used during the SBHO search itself. The selected configuration was then applied to model training on the ARCADE training split of 1,000 images, model selection checking point and early stopping used the ARCADE validation split of 200 images; and all reported results are computed on the disjoint ARCADE test split of 300 images, which was not used at any stage of hyperparameter selection or training.

Candidates are scored analytically through the fitness function in Equation 18 against empirically derived prior targets prior to training. The ARCADE train/validation/test split used for model selection and reporting is described in Section 3.1.7.

The Swarm-Based Hyperparameter Optimiser (SBHO), as shown in Figure 5, automates the selection of sensitive training settings that are difficult to tune manually because of class imbalance and conflicting loss objectives. SBHO searches four parameter spaces simultaneously: loss weights, background class weight, augmentation rotation range, and the Tversky false negative penalty β. The optimisation uses 8 agents, where each agent represents a different parameter configuration. Over 15 iterations, every agent is evaluated using a fitness function that rewards balanced loss behaviour, penalizes deviation from prior target values, and favours empirically validated parameter regions. The best-performing agent then guides the others through a cooperative attraction update with a small Gaussian perturbation to avoid early convergence. After optimisation, the best parameter configuration is directly applied to the training pipeline, removing the need for manual tuning.

**Figure 5:**
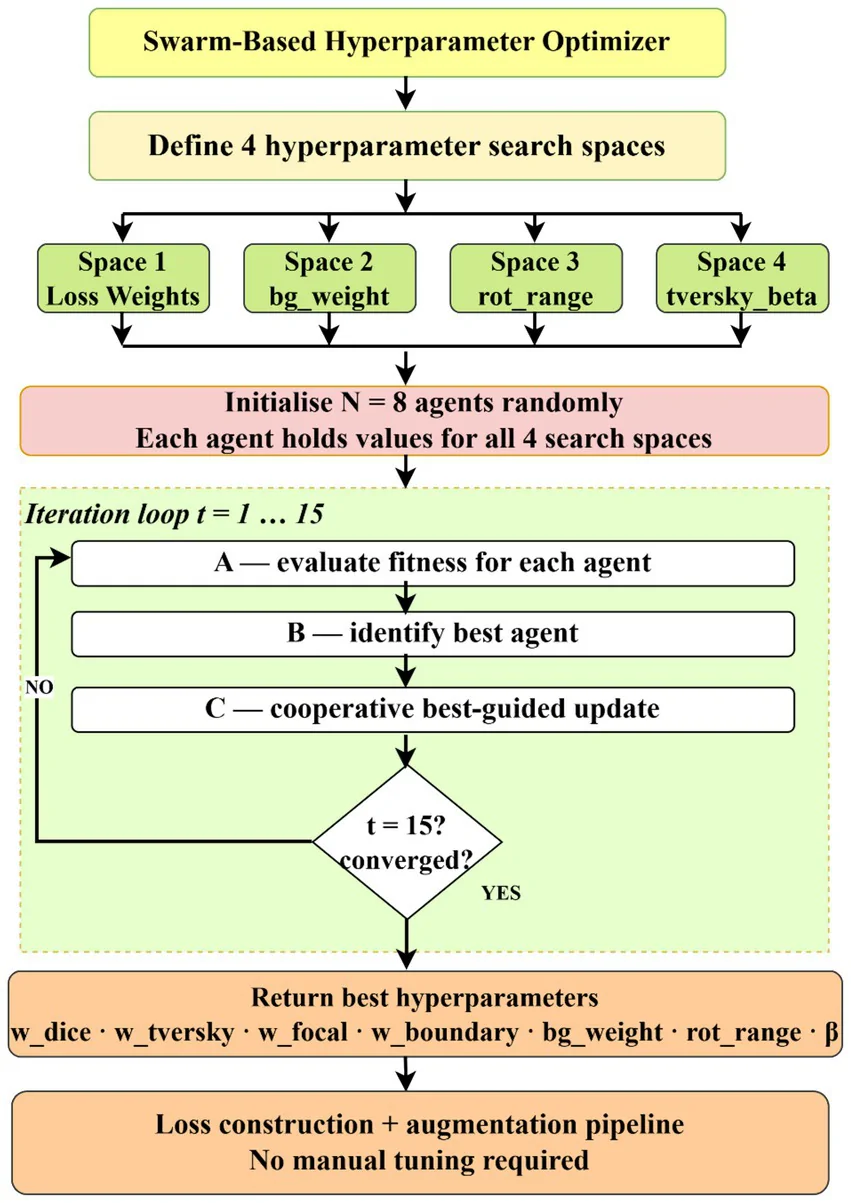
Swarm based hyperparameter optimiser workflow.

#### 3.1.8 Loss function

The stage 1 loss combines four different loss terms as calculated with the use of Equation 20, with weights automatically optimised by SBHO.

$$\begin{array}{l} L_{1}=w_{\text{dice}}.L_{\text{Dice}}+w_{\text{tversky}}.L_{\text{Tversky}}(\beta )\\ +w_{\text{focal}}.L_{\text{focal}}+w_{\text{boundary}}.L_{\text{boundary}}\; \end{array}$$ (20)

Each term targets a segmentation challenge, the dice loss improves the overlap between the predicted output and ground truth, helping to handle class imbalance. The Tversky loss gives a higher penalty to false negatives than false positives, which helps preserve thin and sparse blood vessels. The focal loss reduces the effect of easy background pixels and focuses more on difficult vessel pixels that are wrongly classified. The boundary loss increases the importance of vessel edges using a morphological boundary mask, helping to produce sharper segmentation boundaries.

The total loss is calculated by using Equation 21 from four branches, where each branch is assigned a decreasing weight.

$$L_{\text{total}}=0.55.L_{1}^{\text{main}}+0.20.L_{1}^{\mathrm{aux}1}+0.15.L_{1}^{\mathrm{aux}2}+0.10.L_{1}^{\mathrm{aux}3}$$ (21)

The main branch has the highest contribution, while the auxiliary branches provide extra guidance during training.

#### 3.1.9 Stage 1: binary vessel segmentation

Directly training a 27-class model on raw X-ray angiography images is difficult because most of the image is just background more than 95%. This makes the model focus on background than on small coronary vessels, specifically very thin vessels, which are often missed. The model also spends too much effort trying to separate vessels from noisy background instead of learning different vessel types. To solve this, we use a two-stage approach, in stage 1, the model first finds all vessel regions using a simple binary mask. In Stage 2, another model looks only at these vessel regions and classifies the vessel types. This reduces background noise, improves learning, and helps to preserve thin vessels for accurate detection and classification.

##### 3.1.9.1 Architecture pipeline and optimisation process

In stage 1, architecture is shown in Figure 6 the input X-ray angiography image is resized to 256 × 256 × 3 and passed through an encoder-decoder network designed to accurately detect blood vessels. A pretrained EfficientNetV2S encoder extracts features on multiple scales skip features at four spatial resolutions of 128 × 128, 64 × 64, 32 × 32 and 8 × 8 bottleneck while preserving fine vessel details. To better capture long and connected vessel structures, the model used SS2D block that learns global vessel continuity across the image. During decoding, attention gates help remove background noise from each skip connection before merging with the upsampled decoder output. Four decoder blocks progressively recover spatial resolution through double Convolution, Batch normalisation and ReLU layers with vessel aware AGDrop regularization. Deep supervision uses extra outputs at different image sizes to help the model learn vessel features better at every scale to preserve thin vessels. Before training, the swarm Based Hyperparameter optimiser automatically selects optimal loss weights, background class weight, augmentation rotation range, and Tversky beta removing manual tuning entirely. The network gradually reconstructs the vessel map and finally produces a binary mask showing vessel locations.

**Figure 6:**
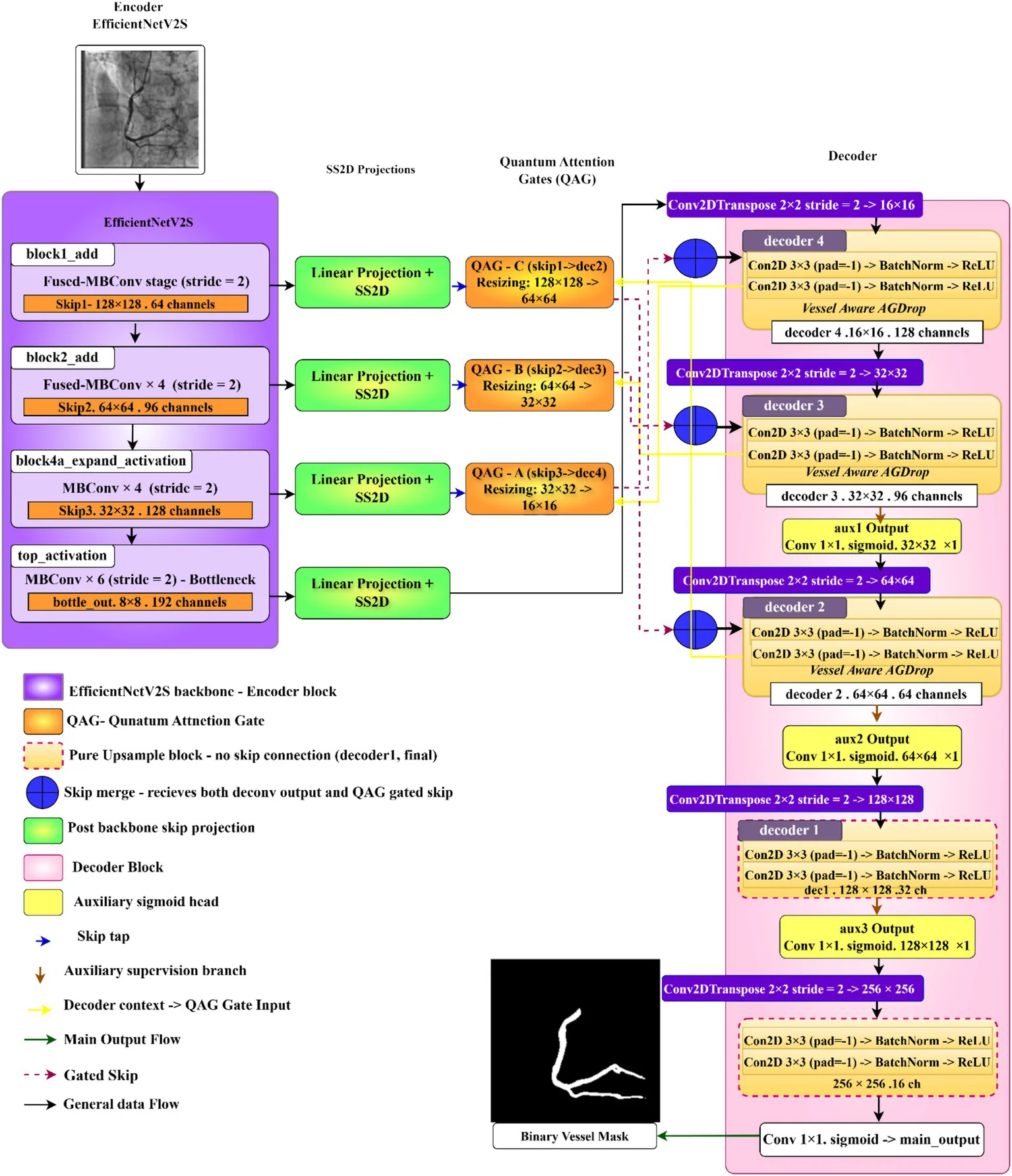
Proposed binary vessel segmentation network for stage 1.

### 3.2 Stage 2: 27-class multi segmentation

Stage 2: 27 class vessel multi vessel segmentation as shown in Figure 7 uses a 4-channel input tensor, $X_{\text{guided}}\in R^{H\times W\times 4}$ , created by combining the preprocessed XCA image of 3 channels with Stage 1 probability map P_1_ in 1 channel. The model then produces a dense 27-class probability map, P_2_ $\in R^{H\times W\times 27}$ . These 27 classes include 26 coronary artery segments defined in the ARCADE SYNTAX annotation protocol and one background class 0.

**Figure 7:**
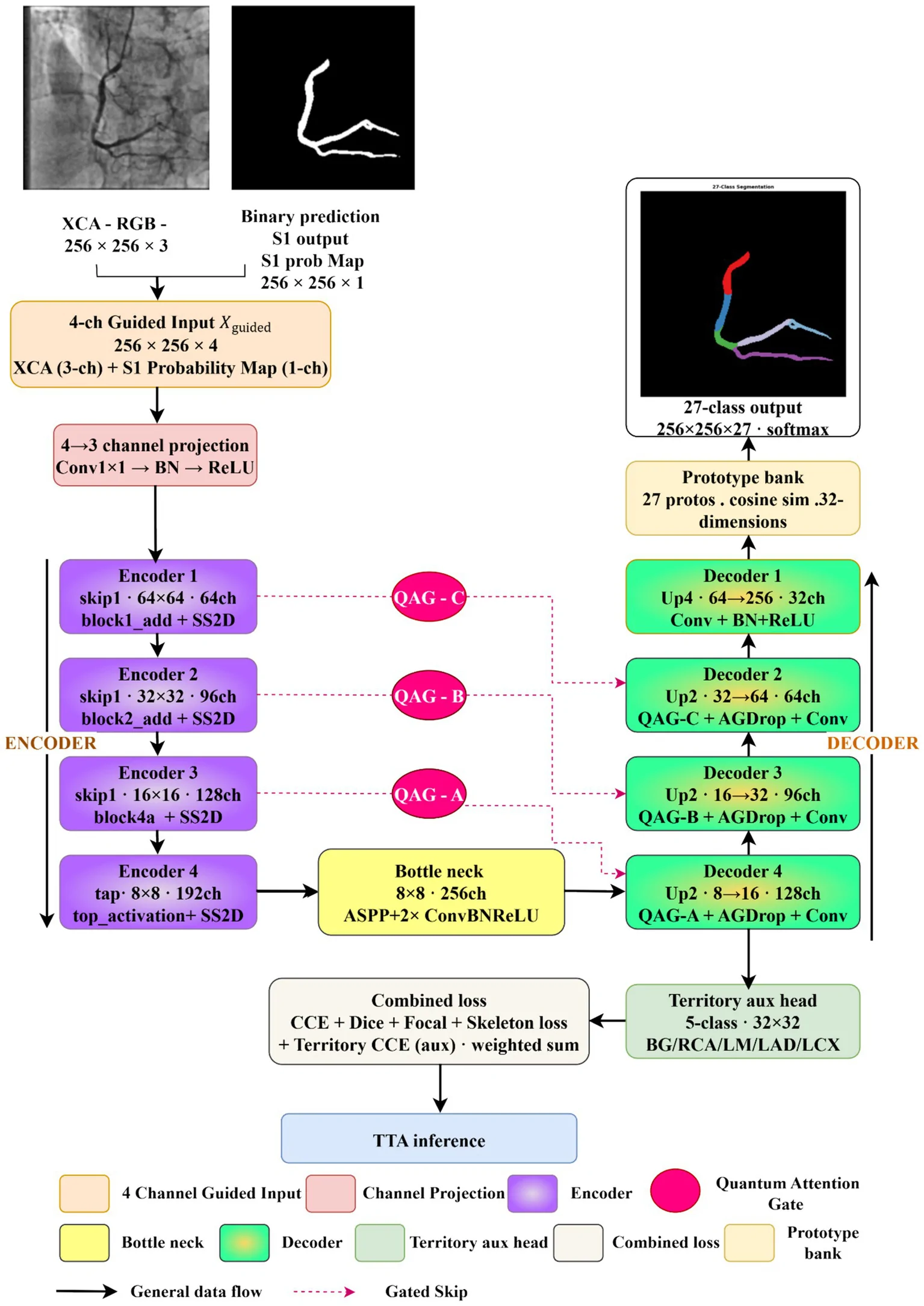
Stage 2: 27-class multi-vessel coronary artery segmentation network.

#### 3.2.1 Four to three channel projection

The EfficientNetV2S backbone was originally trained on ImageNet using standard 3-channel RGB images. Therefore, its first convolution layer expects an input with 3 channels. Since the guided input $X_{\text{guided}}\in R^{H\times W\times 4}$ contains 4 channels, directly using it would require reinitializing the first layer and losing important pretrained features such as edge, texture and contrast filters that are critical for robust vessel boundary detection in angiographic images.

$$X_{\text{proj}}=\text{ReLU}(\mathrm{BN}(\mathrm{Con}v_{1\times 1}\;(X_{\text{guided}}))),X_{\text{proj}}\in R^{\left(256\times 256\times 3\right)}$$ (22)

To avoid this, a lightweight channel projection module in the Equation 22 is added before the backbone. This module uses 1 × 1 pointwise convolution, followed by BN and ReLU activation, to convert the 4-channel input into a compatible 3-channel representation while preserving the pretrained backbone weights. A learnable 1 × 1 convolution is used instead of directly multiplying the image by the Stage-1 vessel-probability map. Direct multiplication can reduce image information in regions where Stage-1 probability is low. Because the 1 × 1 projection instead treats the vessel-probability map as an additional guidance channel rather than a hard mask, the backbone retains full image context and can recover from imperfect Stage-1 predictions rather than being constrained by them. Since the projection is learnable, it can learn how much importance to give to the vessel probability map rather than using a fixed combination rule.

It is also important to note that X-ray coronary angiograms are grayscale images. During preprocessing, the enhanced grayscale image is copied into three identical channels to create the standard 3-channel input. Therefore, these three channels do not contain different colour information. The useful information in the 4-channel guided input comes from the grayscale image and the added Stage-1 vessel-probability map. The 1 × 1 projection then learns how to combine these two types of information and converts them into the 3-channel format required by the pretrained backbone.

#### 3.2.2 EfficientNetV2S feature extractor

The EfficientNetV2S backbone is used as a separate feature extraction model with its own 3-channel input layer. This allows all pretrained ImageNet weights to load correctly without any mismatch issues. The projected input tensor X_Proj_ is passed to this feature extractor, which generates four feature maps for skip connections at resolutions of 64 × 64, 32 × 32, 16 × 16, and 8 × 8, similar to Stage 1.

Training is performed in two phases. In Phase 1 performed (epochs 0–25), the feature extractor is frozen so that only the decoder layers are trained. This helps the decoder learn stable features first. In Phase 2 performed 25–80, the backbone is unfrozen and the entire network is fine-tuned end-to-end using the Adam optimiser with a LR of 2 × 10^−4^ and clip norm of 0.5.

#### 3.2.3 SS2D, QAG, and AGDrop in stage 2

The SS2D, QAG, and AGDrop modules used in Stage 2 have the same structure as in Stage 1, but they use separate trainable weights. Each encoder skip feature is first adjusted to the required number of channels using a Conv 1 × 1, BN and ReLU block and then refined with an SS2D block before being transferred to the decoder. The decoder has five levels. First, the bottle neck feature map 8 × 8, 192 channels, is upsampled to 16 × 16 of 128 channels, in s2_dec4 and combined with skip3 using QAG-A. Next, s2_dec3 up samples to 32 × 32 of 96 channels using skip2 with QAG-B. Then s2_dec4 up samples to 64 × 64 of 64 channels, using skip1 with QAG-C. After each QAG operation, AGDrop regularization and a fusion block consisting of concatenate -Conv of 3 × 3 BN, ReLU and Conv 3 × 3, ReLU are applied. The last two decoder stages, s2_dec1, and s2_final, only perform transposed convolution up sampling without skip connections. Feature map size is to 128 × 128 of 32 channels and finally to 256 × 256 of 16 channels, since the EfficientNetV2s backbone does not provide encoder features at these resolutions.

#### 3.2.4 Component: vessel prototype memory bank

Following the final up sampling stage, a vessel prototype memory bank refines per-pixel features before classification. The bank maintains a trainable embedding matrix $\Pi \in R^{\left(27\times 32\right)}$ , storing one 32-dimensional prototype vector per vessel class. The decoder output F is projected to 32 channels through Conv 1×1 , yielding F_Proj. Both F_Proj and Π are L2 normalized, and cosine similarity is computed between each pixel and all 27 prototypes, as defined in Equation 23.

$$S(i,j,k)=\hat{F}\;\text{Proj}(I,J).{\hat{\pi }}_{k},S\;\epsilon \;R^{\left(256\times 256\times 27\right)}$$ (23)

Temperature scaled SoftMax with scale of 10 changes similarity scores into attention weights. These weights are then converted back to C channels and added to the original features using a residual connection and layer normalisation, as expressed in Equation 24.

$$F_{\text{out}}=\text{Laye}r\;\text{Norm}(F+\mathrm{Con}v_{1\times 1}\;(\text{SoftMax}(10.S)))$$ (24)

This method helps the model learn clear feature representations for each vessel class by connecting them to specific prototype features. As a result, the network can better identify rare and small vessel branches, such as D2a and OM2a, even though these classes appear with very few pixels during training.

#### 3.2.5 Component: territory auxiliary supervision

Three auxiliary heads are added at different decoder resolutions to predict 5 coarse coronary territories such as background, RCA, LM, LAD, and LCX. The territory labels are automatically generated from the 27-class annotations. This supervision is used to improve gradient flow and speed up training, and help the network first learn the overall coronary anatomy before learning fine vessel segments and reduce major anatomical mistakes, such as predicting vessels in the wrong coronary territory.

#### 3.2.6 Stage 2 loss function

The Stage 2 model uses a combination of cross-entropy, dice, focal, and skeleton loses, along with auxiliary territory supervision as calculated by using Equation 25.

$$\begin{array}{l} L_{2}=w_{\mathrm{cce}}L_{\mathrm{CCE}}+w_{\text{dice}}L_{\text{Dice}}+w_{\text{focal}}\;L_{\text{focal}}\\ +w_{\text{skel}}L_{\text{skeleton}}+w_{\mathrm{aux}}\;L_{\text{Territory}} \end{array}$$ (25)

#### 3.2.7 Test time augmentation

During inference, test-time augmentation (TTA) was applied to reduce the variance of the predicted probability maps. Each test image was transformed under five grid-preserving symmetries of the square: the identity, horizontal flip, vertical flip, combined horizontal-vertical flip (a 180° rotation), and transpose (reflection across the main diagonal). The model generates a prediction for each transformed image, each prediction is mapped back to the original orientation by the corresponding inverse transform, and the resulting probability maps are averaged as described in Equation 26. Because every transform is an exact pixel permutation, the averaging introduces no interpolation and preserves thin-vessel geometry through the transform and inverse cycle. TTA is applied here as a variance-reduction step rather than as a model of image acquisition: the transformed images need not correspond to clinically realizable orientations, since only the inverse-mapped, averaged prediction is evaluated. Averaging these consistent but decorrelated predictions stabilizes the output at thin, low-contrast distal vessel segments, where single-pass predictions are least certain.

$$\overset{`}{y}=\text{argma}x_{k}\;\frac{1}{5}\;\sum_{a=0}^{4}\mathrm{au}g_{a}^{-1}\;(P_{2}^{\left(a\right)}\;)$$ (26)

As shown in the Figure 8, averaging over these transforms produces a smoother, more continuous probability map. It removes small, scattered false responses along thin vessels, so the final segmentation is closer to the ground truth than a single prediction.

**Figure 8:**
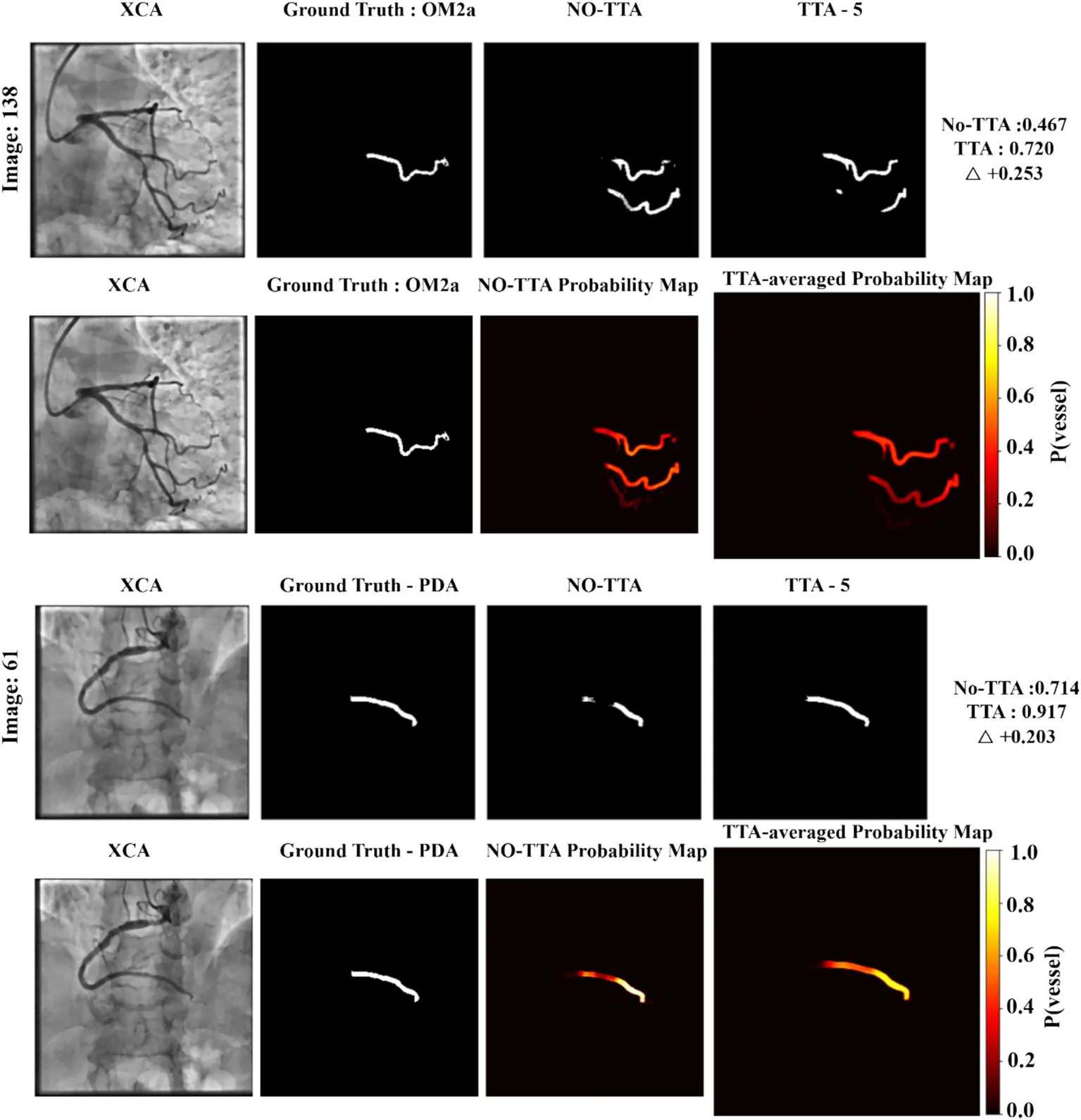
Effect of test-time augmentation on thin vessel segmentation.

### 3.3 Stage 3: post processing pipeline centreline extraction and stenosis quantification

The 27-class segmentation map from Stage 2 identifies each coronary artery segment at the pixel level. However, stenosis analysis also requires the vessel centreline and vessel width. The centreline provides a one-pixel-wide path of the vessel, which is important because stenosis is measured along the vessel direction. Therefore, Stage 3 extracts vessel centrelines from the predicted segmentation map using morphological skeletonization, applies morphological opening and closing to remove noise before skeletonization, and sorts the resulting skeleton points in proximal to distal order using nearest neighbor traversal initialized from the point closest to the image origin, which serves as a proxy for the anatomical proximal end. The ordered centreline is then used as the basis for all subsequent width measurement and stenosis estimation.

#### 3.3.1 Perpendicular ray-based vessel width measurement

Traditional distance transform methods estimate vessel width using the shortest distance to the background, but this often gives smaller and inaccurate widths for curved vessels. To address, this, a perpendicular ray-casting method is used, where rays are projected across the vessel at each centreline point to measure the true vessel width in the correct anatomical direction. At each centreline pixel, the local vessel tangent t = ( $t_{x}$ , $t_{y}$ ) is estimated using finite differences over neighboring centreline points. The perpendicular direction n is then computed as $n=(-t_{y}.t_{x}$ ).

From each centreline point, rays are extended in both perpendicular directions up to a maximum distance of 40 pixels, stopping when the ray exists. The vessel width at the point is the sum of the two ray lengths plus the central pixel. The resulting width profile is smoothed using a Savitzky–Golay filter of window = 7, polynomial order = 3 to reduce noise while preserving the shape of stenotic narrowing’s. This approach gives more accurate width measurements for curved and thin vessels than traditional distance transform methods. This approach yields more accurate width measurements for curved and thin vessels than distance-transform methods. As the ARCADE SYNTAX subset provides vessel-region annotations without reference lumen diameters, width measurement is characterized by its stability under segmentation error, quantified in section 5.5 by comparing width profiles computed from predicted versus ground-truth vessel masks.

#### 3.3.2 Aquila optimiser for minimum lumen localisation

Parameter diameter stenosis is determined by the minimum lumen width along each coronary vessel. Selecting this minimum directly from the raw width profile is sensitive to single-pixel noise and skeletonization artefacts, which may displace the reported lesion site. To address this, an Aquila Optimiser refines the minimum-lumen position p* on the smoothed width profile using the objective f(p) = |D(p) – D_min, raw_| + λ_AQ._ | ▽D(p)|, which favours positions at a genuine local width minimum with low gradient rather than at the profile noise. The optimiser combines expanded exploration and narrowed exploitation strategies with Levy-flight perturbations, using 15 agents over 30 iterations. This procedure is unsupervised; it operates solely on the geometry of the extracted width profile and requires no lesion annotations. This clinical threshold of 50% diameter reduction defined by the SYNTAX methodology (Sianos et al., 2005) is applied uniformly across all vessel classes.

#### 3.3.3 Present class dice

Standard mean-Dice treats anatomically absent vessel classes as segmentation failures, which unfairly penalises the model. If a patient does not have a Ramus vessel, correctly predicting background should not reduce the score. To solve this, the proposed present-class dice calculates Dice only for vessel classes that are actually present in the ground-truth mask which supports ≥ 50 pixels, as calculated by using Equation 27.

$$\mathrm{Dic}e_{\text{present}}=\frac{1}{|C_{p}|}\;\sum_{c\in C_{p}}\mathrm{Dic}e_{c}$$ (27)

Where $C_{p}$ represents the set of vessel classes present in the image. This metric provides a fairer evaluation for highly imbalanced coronary datasets, especially for rare vessel classes such as OM1, D2a, and Ramus.

#### 3.3.4 Proximal reference diameter for clinical QCA alignment

The reference vessel diameter is calculated using the 75th percentile diameter from the proximal 20% and the distal 20% of the centreline, linearly interpolated along the vessel to account for physiological tapering following the clinical QCA standard used in TIMI and SYNTAX studies. The 75th percentile is used to reduce the effect of noise near vessel endpoints. This helps the automatic measurement better match clinical catheterisation laboratory evaluations and supports standard stenosis evaluation guidelines. Percent diameter stenosis at each centreline point is then computed by utilising the Equation 28

$$\%\mathrm{DS}=(1-\frac{D(p^{*})}{D_{\mathrm{ref}}\;(p*)}\;)\times 100$$ (28)

Where, $D(p^{*})\;$ is the local vessel diameter at centerline point $\;P^{*}$ and $D_{\mathrm{ref}}\;(p*)\;\;$ is the interpolated reference diameter at that point. This formulation directly mirrors clinical quantitative coronary angiography and supports comparison with catheterisation-laboratory evaluations.

### 3.4 Stage 4 and 5: stenosis grading and SYNTAX score estimation

The SYNTAX score is calculated automatically from the detected stenosis results. Vessel calibre is measured in image pixels, as the ARCADE dataset does not provide per-image DICOM calibration factors. The 1.5 mm inclusion criterion is therefore applied at the segment level rather than by absolute measurement. The 26 coronary segments annotated in ARCADE follow the SYNTAX segment definition (Sianos et al., 2005), under which only vessels of ≥ 1.5 mm calibre are assigned a segment and scored. Annotation is therefore determined by membership of these named segments rather than by vessel width alone. A visible branch that does not correspond to one of the defined SYNTAX segments is not annotated, even if its calibre exceeds 1.5 mm. A lesion localised to any annotated segment consequently satisfies the calibre criterion by construction. Each lesion with ≥ 50% diameter narrowing contributes to the total score according to the anatomical weight of its segment and its lesion multiplier (Sianos et al., 2005). Non-occlusive lesions (50–99%) receive a multiplier of 2, and total occlusions a multiplier of 5. Lesions below 50% are recorded by the pipeline but do not contribute to the score. All lesion scores are summed to obtain the final SYNTAX score. Patients are then grouped into three risk categories: LOW risk (≤22), Intermediate risk (23–32) and HIGH risk (≥ 33), which helps guide treatment decision support. The system reduces the need for time-consuming manual scoring and provides fast and reproducible evaluation. However, the current method uses single-view XCA images, whereas clinical SYNTAX scoring is usually performed using multiple angiographic views.

### 3.5 Algorithm

ALGORITHM 1 **VMamba-QAG-Net End-to-End Pipeline for coronary SYNTAX score estimation.**

**Figure:**
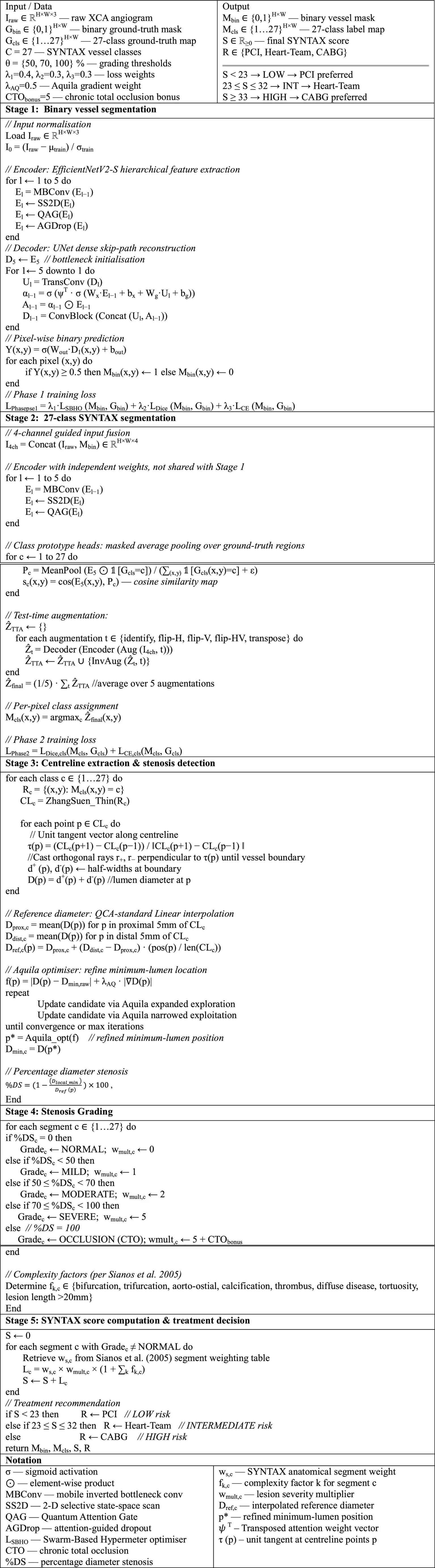

## 4 Experimental setup

### 4.1 Dataset preparation

The ARCADE dataset (Mendeley, 2025; Zenodo, 2023) contains 3,000 X-ray coronary angiography (XCA) images acquired from patients suspected of having CAD at the Research Institute of Cardiology and Internal Diseases, Almaty, Kazakhstan. The collected images are used Philips Azurion 3 and Siemens Artis Zee angiography systems with a resolution of 512 ⨯ 512 pixels. During image collection, patients were positioned in a supine position, and heart rates were maintained between 70–85 beat per minute (bpm) using beta-blockers to reduce motion artifacts, Contrast agents such as Omnipaque were injected through femoral artery access to improve visualization of the coronary arteries. The images were captured from six standard viewing angles, including four views for the left coronary artery and two views for the right coronary artery. For each patient, around 300–800 angiographic frames were initially recorded. From these, up to two high-quality frames per viewing angle were selected based on better contrast visibility, fewer motion artifacts, and clearer stenosis appearance, resulting in a maximum of 12 images per patient. All images were annotated using CVAT and carefully verified by expert cardiologists through multiple validation stages to ensure accurate pixel-level annotations. The dataset is divided into two subsets: the Stenosis subset and the SYNTAX subset, each containing 1,500 XCA images. Both subsets are split into 1,000 training images, 200 validation images, and 300 testing images. In this work, only the SYNTAX subset is used, as its 26-segment anatomical annotations provide the detailed vessel labelling required for centreline extraction and segment-wise SYNTAX scoring, which the stenosis subset, a disjoint set of images annotated only for plaque regions, does not provide. Because the SYNTAX subset does not include per-lesion stenosis ground truth, the rule-based Stage 3–5 stenosis analysis is derived geometrically from the predicted segmentation and is characterized by its geometric output and population-level score distribution rather than validated against reference lesions.

### 4.2 Implementation details

Hyperparameters and configuration details are detailed in Table 4 for all training stages on the ARCADE dataset. Stage 1 binary vessel segmentation, stage 2 27-multiclass segmentation, stage 3 rule-based stenosis detection and SYNTAX score estimation. Our proposed VMamba-QAG-Net approach is executed on Python language, Tensor Flow CPU with a 12th Gen Intel^®^ Core (TM) i9- 12900KF, an NVIDIA GeForce RTX 3090 GPU, and 32 GB of system memory.

**Table 4:** Implementation details of VMamba-QAG-Net.

Setting	Stage 1 Binary segmentation	Stage 2 27-class segmentation	Stage 3 Stenosis detection and SYNTAX score
Preprocessing pipeline			
Denoising	Bilateral Filter (d = 7, $\sigma_{\text{color}}=50,\sigma_{\text{space}}=50)$		
Contrast/sharpening	CLAHE (clip limit = 3.0, tile grid = 8×8 ). Unsharp mask (α = 1.2, β = −0.2, σ = 1.0)		
Normalisation/input size	÷ 255 → [0,1]. 256×256×3	÷ 255 → [0,1]. 256×256×4	Rule based
Model Architecture			
Backbone	EfficientNetV2-S	EfficientNetV2-S Independent weights not shared with Stage 1	-
Encoder initialization	ImageNet pre-trained backbone frozen in Phase 1, finetuned in Phase 2	ImageNet pre-trained fully trainable from initialization no weight sharing with Stage 1	-
Output classes/bottleneck	2/192 ch	27/192 ch	-
Decoder channels	192 → 96 → 64 → 32 → 16		-
Skip levels/SS2D dirs./ QAG	4 skip levels (E1-E4) E5 used as bottleneck D5/ SS2D: 5 directions (H, V, diagonal d = 1, d = 2, d = 4) /QAG: Filters ÷ 2		-
Prototype bank/AGDrop rates	-	27 Prototypes (one per vessel class)/0.20,0.15, 0.10, 0.05	-
Deep supervision/parameters	4 outputs: (main+ 3 auxiliary)		Rule based
Training configuration			
Batch Size/Epochs	16/100	16/80	-
Phase 1 -frozen (ep. LR)	30 eps. le^−3^	25 eps. le^−3^	-
Phase 2 -full fine tune (ep. Peak LR)	70 eps. 2e^−4^	55 eps. 2e^−4^	-
Optimiser	Adam		
Loss functions			
Combined loss components (S1)/(S2)	SBHO-weighted combination of: Dice + Tversky + Focal + Boundary	Weighted CCE + Dice + Focal+ Skeleton	-
Weights/ key parameters	0.35/0.40/0.15/0.10	0.30/0.35/0.20/0.15	-
Tversky β/Focal γ, α (S1)	Β = 0.82, γ = 2.5, α = 0.85	-	
Focal γ (S2)	-	γ = 2.5	
SBHO	8 agents, 15 iterations	-	-
Deep supervision weights	Main: 0.55. Aux1 0.20. Aux2 0.15. Aux3 0.10		-
Auxiliary supervision target	Binary Vessel	Territory map (5-class)	-
Data augmentation			
Horizontal/Vertical flip	*p* = 0.5 (each)		-
Random rotation	*p* = 0.5. ±20°	*p* = 0.5. ±15°	-
Brightness jitter	*p* = 0.5. scale 𝜖 [0.85, 1.15]		-
Test-time augmentation (TTA)	-	5 transforms	-
TTA transforms	-	Original. H-flip.V-flip. HV-flip. Transpose	-
Stage 3 – Stenosis quantification & SYNTAX estimation			
Centreline and width	-	-	Morphological skeletonization + perpendicular ray-casting (max 40 px); S-G smooth (w = 7, *p* = 3)
Percent diameter stenosis (%DS)	-	-	$\%\mathrm{DS}=(1-\frac{\left(D_{\text{local}\_\min}\;\right)}{D_{\mathrm{ref}}\;(p)}\;)\times 100$
Threshold optimisation	-	-	Aquila 15 agents 30 iterations performed on validation set only (200 images). Thresholds fixed thereafter, applied without modification at test-set evaluation.
SYNTAX scoring risk tiers	-	-	LOW risk (≤22) – PCI preferred, Intermediate risk (23–32)- Heart-Team and HIGH risk (≥33)—CABG preferred (AHA/ACC guidelines)

### 4.3 Evaluation metrics

Segmentation performance was evaluated using Equations 29–34, 36 are listed as Dice coefficient, IoU, Recall, Precision, Accuracy, specificity and centerline Dice (clDice). For multi class evaluation, the present class dice metric was also used to fairly measure performance for vessel classes that may be absent in some images. Stage −3 clinical results were evaluated using percentage diameter stenosis, total SYNTAX score Equation 35, compared with reported SYNTAX trial ranges.

$$\text{Dice Coefficient}=\frac{\left(2\times |\mathrm{GT}\cap \mathrm{PO}|\right)}{\left(|\mathrm{GT}|+|\mathrm{PO}|\right)}$$ (29)

$$\text{Jaccard Index}\;(\mathrm{IoU})=\frac{\left(|\mathrm{GT}\cap \mathrm{PO}|\right)}{\left(|\mathrm{GT}\cup \mathrm{PO}|\right)}$$ (30)

$$\text{Accuracy}=\frac{\left(\mathrm{TP}+\mathrm{TN}\right)}{\left(\mathrm{TP}+\mathrm{TN}+\mathrm{FP}+\mathrm{FN}\right)}$$ (31)

$$\text{Precision}=\frac{\mathrm{TP}}{\left(\mathrm{TP}+\mathrm{FP}\right)}$$ (32)

$$\text{Recall}=\frac{\mathrm{TP}}{\left(\mathrm{TP}+\mathrm{FN}\right)}$$ (33)

$$\text{Specificity}=\frac{\mathrm{TN}}{\left(\mathrm{TN}+\mathrm{FP}\right)}$$ (34)

$$\text{SYNTA}X_{\text{total}}=\sum_{c\in A}W_{c}\times M_{s}^{\left(c\right)}$$ (35)

$$\text{clDice}=2.\frac{(T_{\text{prec}}.T_{\text{sens}})\;}{\left(T_{\text{prec}}+T_{\text{sens}}\right)}$$ (36)

$T_{\text{prec}}$ is the topology precision, which shows how much of the predicted centerline is inside the ground-truth mask. $T_{\text{sens}}$ is the topology sensitivity, which shows how much of the ground-truth centerline is covered by the predicted mask. clDice measures how well the segmented vessels preserve their connectivity by comparing the vessel centerlines instead of only comparing pixel overlap.

## 5 Results

### 5.1 Comparison methods

Table 5, presents a comparative evaluation of the proposed VMamba-QAG-Net against State of The Art (SOTA) coronary angiogram segmentation methods, with all methods evaluated on the same ARCADE dataset. In binary segmentation, it obtained a Dice coefficient of 0.9070, Jaccard Index of 0.8294, accuracy of 0.9841, precision of 0.8950, recall of 0.9210, and specificity of 0.9921 on the 1,500-image SYNTAX subset. Although some existing methods reported slightly higher results for individual metrics, none attained such consistent performance across all measures. These results show the effectiveness of the VMamba-based architecture in handling complex vessel structures and anatomical variations. In multi-class segmentation, the model attained a Dice coefficient of 0.8214, indicating robust performance despite the challenges of vessel boundary ambiguity. These findings confirm the reliability and clinical potential of VMamba-QAG-Net for both binary and multi-class coronary artery segmentation.

**Table 5:** Performance evaluation of the proposed VMamba-QAG-Net relative to SOTA techniques.

Work	Number of angiograms	Method	Dice coefficient/Present dice coefficient	Jaccard index	Accuracy	Precision	Recall	Specificity
**Proposed work-binary segmentation**	**1,500**	**Proposed VMamba-QAG-Net (binary segmentation)**	**0.9070**	**0.8294**	**0.9841**	**0.8950**	**0.9210**	**0.9921**
**Proposed work-multi class segmentation**	**1,500**	**Proposed VMamba-QAG-Net 27-class multi segmentation**	**0.7512/*0.8214***	**0.6018**	**0.9514**	**0.7248**	**0.7134**	**0.9763**
Sun et al. (2026)	NA	MPF + Pseudo-stenosis pretraining	60.52	-	99.22	-	62.24	99.58
Yang et al. (2026)	1,000	UNet	84.43% across 20 coronary segments	72.69	98.41	90.96	78.79	99.23
Lalinia et al. (2026)	3,000	Swin UNETR (Fine-tuning with label smoothing)	0.5375	-	0.8025	0.5577	0.6099	0.5375
	3,000	Swin UNETR (Adapter-tuning with label smoothing)	0.5519	-	0.8089	0.5988	0.5860	0.5519
Zhao et al. (2025)	1,500	MACAN phase 1/phase 2	0.4287/0.4126	-	-	-	-	-
Chen et al. (2025)	1,500	SFAGDeepLabv3+	91.56	85.41	98.90	91.05	92.07	-
Ferrari et al. (2025)	1,500	CoroSAM bottleneck 25%	0.8719	-	-	0.8562	0.8941	-

Note: Bold values denote the results of the proposed VMamba-QAG-Net method.

### 5.2 Ablation study

Binary segmentation performance improved progressively as additional components were added in the ablation study as detailed in Table 6. The baseline model attained a Dice Score of 0.7124. Adding SS2D increased the Dice score to 0.7886, showing that it is the most important feature extraction component. QAG and SBHO Loss also improved performance, while AGDrop alone provided only a small gain. The combination of SS2D and QAG attained Dice score of 0.8398, and adding AGDrop further increased it to about 0.8780. The complete model produced the best results, achieving a Dice Score of 0.9060, IoU of 0.8261, and accuracy of 0.9867. The model further attained a clDice of 0.8892 on the ARCADE test set, confirming that the segmented vessels preserve the connectivity of the ground-truth vessel tree.

**Table 6:** Ablation study on binary segmentation component wise performance analysis of the proposed model.

Model variant	SS2D	QAG	AGDrop	SBHO loss	Dice	IoU	Accuracy	Precision	Recall	Specificity
Baseline (EfficientNetV2S + UNet)	✗	✗	✗	✗	0.7124	0.5537	0.9312	0.6981	0.7298	0.9541
+SS2D	✓	✗	✗	✗	0.7886	0.6651	0.9536	0.7591	0.8051	0.9624
+QAG only	✗	✓	✗	✗	0.7598	0.6145	0.9493	0.7303	0.7714	0.9697
+AGDrop only	✗	✗	✓	✗	0.7301	0.5824	0.9354	0.7141	0.7484	0.9561
+SBHO loss only	✗	✗	✗	✓	0.7618	0.6264	0.9412	0.7698	0.7712	0.9664
+SS2D + QAG	✓	✓	✗	✗	0.8398	0.7324	0.9624	0.8284	0.8540	0.9752
+SS2D + QAG + AGDrop	✓	✓	✓	✗	0.8784	0.7821	0.9723	0.8432	0.8814	0.9882
Full Model	✓	✓	✓	✓	0.9060	0.8261	0.9867	0.8950	0.9210	0.9823

The multi class segmentation ablation study as illustrated in Table 7 showed a similar consistent performance improvements as additional components were introduced. The baseline model attained a dice score of 0.5841. SS2D, QAG, and the prototype bank individually enhanced the dice score to 0.6413, 0.6295, and 0.6514, respectively. Combining these components resulted in further performance gains. The full model achieved the best results, with a Dice score of 0.7515, Present Dice of 0.8214, and accuracy of 0.9514, demonstrating that all four components work together effectively to enhance segmentation accuracy.

**Table 7:** Ablation study on multi-class segmentation incremental contribution of each component to overall performance.

Model variant	SS2D	QAG	AGDrop	SBHOLoss	Dice	Present dice	IoU	Accuracy	Precision	Recall	Specificity
Baseline (EfficientNetV2S + UNet)	✗	✗	✗	✗	0.5841	0.6412	0.4623	0.8914	0.5714	0.5512	0.9312
+Stage 1 guidance channel	✓	✗	✗	✗	0.6124	0.6714	0.4192	0.9041	0.5983	0.5820	0.9412
+SS2D only	✗	✓	✗	✗	0.6413	0.7016	0.5218	0.9148	0.6215	0.6128	0.9484
+QAG only	✗	✗	✓	✗	0.6295	0.6911	0.5091	0.9116	0.6112	0.6014	0.9465
+Proto Bank only	✗	✗	✗	✓	0.6514	0.7124	0.5312	0.9198	0.6384	0.6214	0.9524
+SS2D + QAG	✓	✓	✗	✗	0.6841	0.7412	0.5598	0.9284	0.6612	0.6514	0.9612
+SS2D + QAG + proto-Bank	✓	✓	✓	✗	0.7214	0.7821	0.5912	0.9384	0.6984	0.6912	0.9684
Full Model	✓	✓	✓	✓	0.7515	0.8214	0.6018	0.9514	0.7248	0.7134	0.9765

### 5.3 Stage 1: binary segmentation

Figure 9 illustrates binary vessel segmentation results for representative XCA images. Most coronary vessel pixels are correctly detected, indicating accurate vessel localization. False-positive regions are minimal and mainly confined to peripheral regions, while false-negative regions occur primarily at thin distal vessel ends. These results show that stage 1 effectively separates vessels from the background while preserving both major and small vessel branches. The resulting vessel mask guides Stage 2 to classify the detected vessel pixels into 27 classes (the 26 SYNTAX coronary segments plus background), with minimal background interference. The ground-truth masks in Figure 9 are the expert annotations provided with the ARCADE dataset, which were produced and verified by cardiologists and used in this study without any modification. In ARCADE, these annotations label only the 26 coronary segments defined by the SYNTAX methodology. A visible branch that does not correspond to one of these segments is therefore absent from the ground truth by the dataset protocol rather than through segmentation error. Because the SYNTAX subset provides segment labels but no per-lesion stenosis ground truth, any narrowing on such a non-defined branch is likewise not present in these masks.

**Figure 9:**
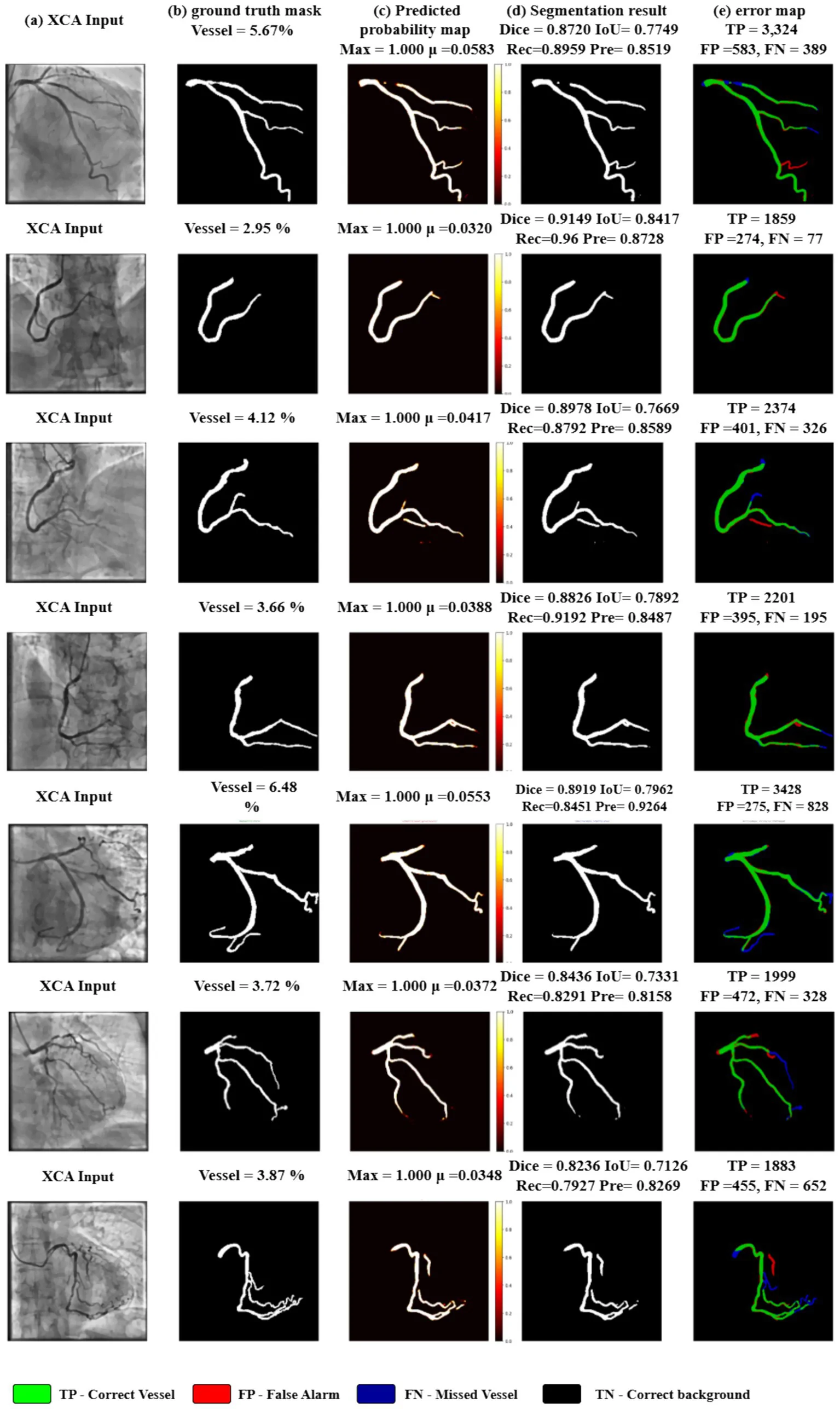
Predicted binary segmentation vessel masks in X-ray coronary angiographic images **(a)** XCA input; **(b)** ground-truth mask; **(c)** predicted probability map (heatmap); **(d)** segmentation result with Dice, IoU, recall, and precision; **(e)** error map (green = TP, blue = FP, red = FN). Ground-truth masks are unmodified ARCADE expert (cardiologist- verified) annotations, which label only the 26 defined SYNTAX segments, visible branches that are not among these segments are not annotated by the dataset protocol.

### 5.4 Stage 2: 27-class segmentation

The Figure 10 shows the predicted 27-class coronary artery segmentation results for representative XCA images. The proposed Stage 2 network accurately labels major coronary arteries and their branches, preserving vessel continuity and correctly identifying both large and small vessel segments. This stage is important because it assigns each vessel pixel to its correct anatomical segment. The resulting 27-class mask enables Stage 3 to perform segment-specific centreline extraction and stenosis analysis, supporting accurate lesion localization and SYNTAX score calculation. Minor boundary overprediction can occur at stenotic sites, particularly on smaller distal vessels. Its effect on the geometric measurements is quantified in Section 5.5, where the mean absolute difference in percentage diameter reduction between predicted and ground-truth masks is 2.8–6.5% in Table 8. This overprediction does not change the resulting stenosis grade in the most segments, with 95.14% severity-grade agreement.

**Figure 10:**
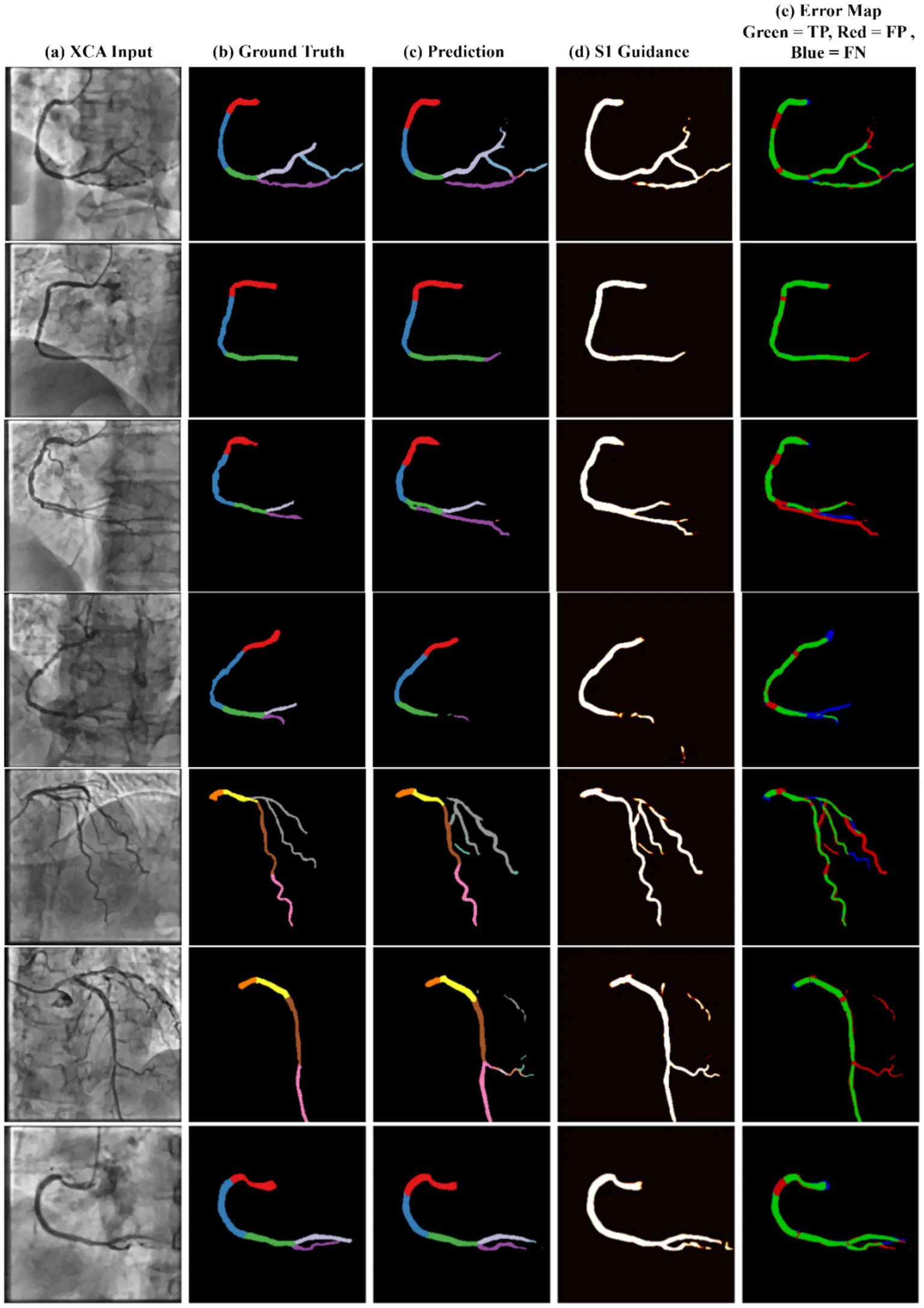
Predicted 27-class coronary artery segmentation outputs on XCA images. **(a)** Input image; **(b)** ground truth mask (27-class color-coded); **(c)** predicted segmentation (27-class color-coded); **(d)** S1 (stage 1) guidance map; **(e)** error map (green = TP, red = FP, blue = FN).

**Table 8:** Stage 3 quantitative outcome on the 300-image ARCADE SYNTAX test set.

Metric	Value
Test images processed	300
Images with ≥ 1 successfully extracted centreline	294 (98.0%)
Vessel segments analysed	1,420 (4.73 ± 1.2 per image)
Segments meeting minimum length (≥ 20 px)	1,363 (96.0%)
Mean centreline length	84.2 ± 31.5 px
Mean reference diameter D__ref_prox_	9.6 ± 1.8 px
Mean distal/ branch reference diameter D__ref_dist_	3.8 ± 0.9 px
Ray-casting success rate (rays terminating <40 px)	94.8%
Segments with %DS ≥ 50s	312 (22.0%)
Mean |Δ%DS|, proximal segments	2.8% ± 1.4%
Mean |Δ%DS|, distal/branch segments	6.5% ± 3.1%
Severity-grade agreement	95.14% (accuracy)
Mean stage 3 runtime per image	0.68 ± 0.12 s

### 5.5 Stage 3: stenosis quantification and SYNTAX score estimation

Stage 3 applied a fully rule-based post -processing pipeline on the stage 2 segmentation output, without requiring any additional model training. It was evaluated on the 300 test images of the ARCADE SYNTAX subset. Since this subset provides 26-segment anatomical annotations but no per-lesion stenosis ground truth, the stenosis analysis is characterized quantitatively, through the processing yield, geometric measurements, and measurement stability reported in Table 9. Qualitatively, through the centreline, ray-casting, severity, and SYNTAX-score visualizations in Figures 11–15, and indirectly, through the population-level SYNTAX score distribution reported in Section 5.6, which is consistent with published SYNTAX trials. Together, these confirm that the pipeline produces anatomically plausible stenosis localizations and clinically reasonable SYNTAX scores.

**Table 9:** Distribution of coronary artery segments across stenosis severity grades.

Severity grade	Stenosis (%DS)	Segments (*n*)	Proportion (%)
Normal/mild	<50%	1,051	74.0%
Non-occlusive lesion	50–99%	267	18.8%
Total occlusion	100%	45	3.2%
Not assessed (<20 px)	-	57	4.0%
Total	-	1,420	100.0%

**Figure 11:**
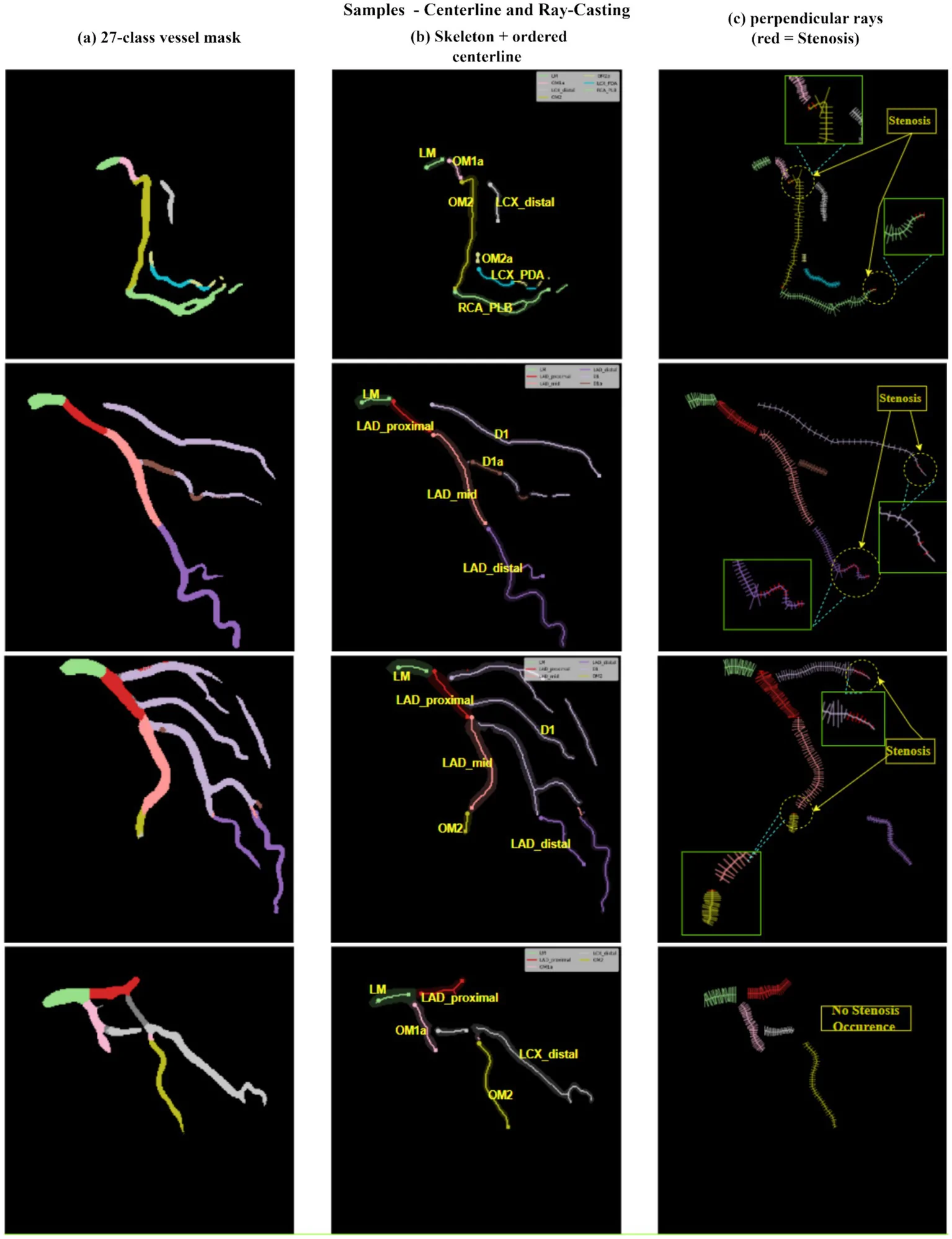
Centreline extraction and perpendicular ray casting for stenosis detection on predicted 27-class vessel masks **(a)** 27-class vessel mask, **(b)** skeleton and ordered centreline with anatomical branch labels, **(c)** perpendicular rays along the centreline, with magnified insets of stenotic regions.

**Figure 12:**
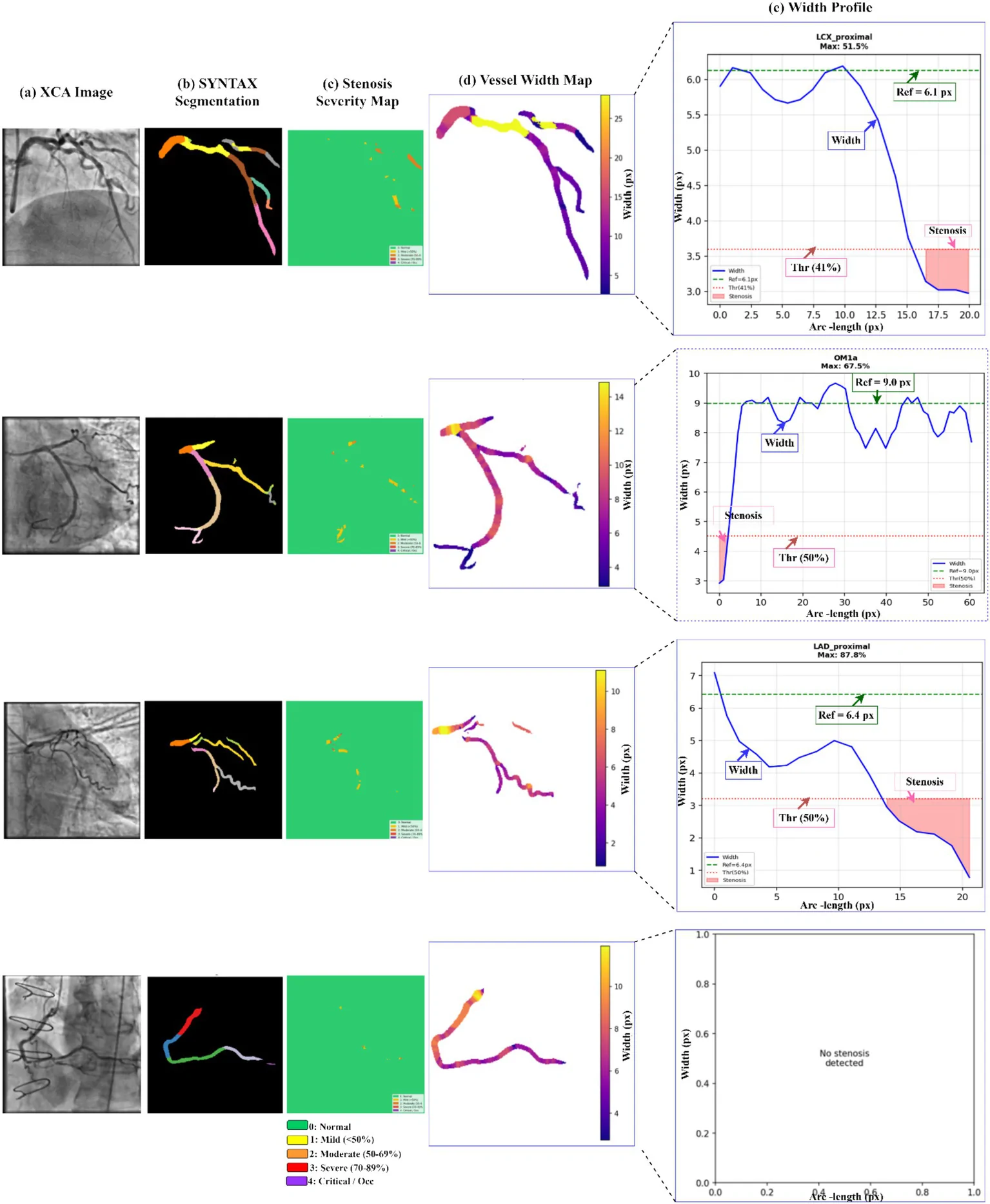
Qualitative stenosis severity and vessel width analysis on XCA images. **(a)** XCA image: input angiography frame, **(b)** SYNTAX segmentation: per-segment coronary artery classification, **(c)** stenosis severity map: severity classification along the vessel, **(d)** vessel width map: local vessel diameter along the centreline, **(e)** width profile along arc: diameter (px) vs. arc-length (px), with reference and threshold lines.

**Figure 13:**
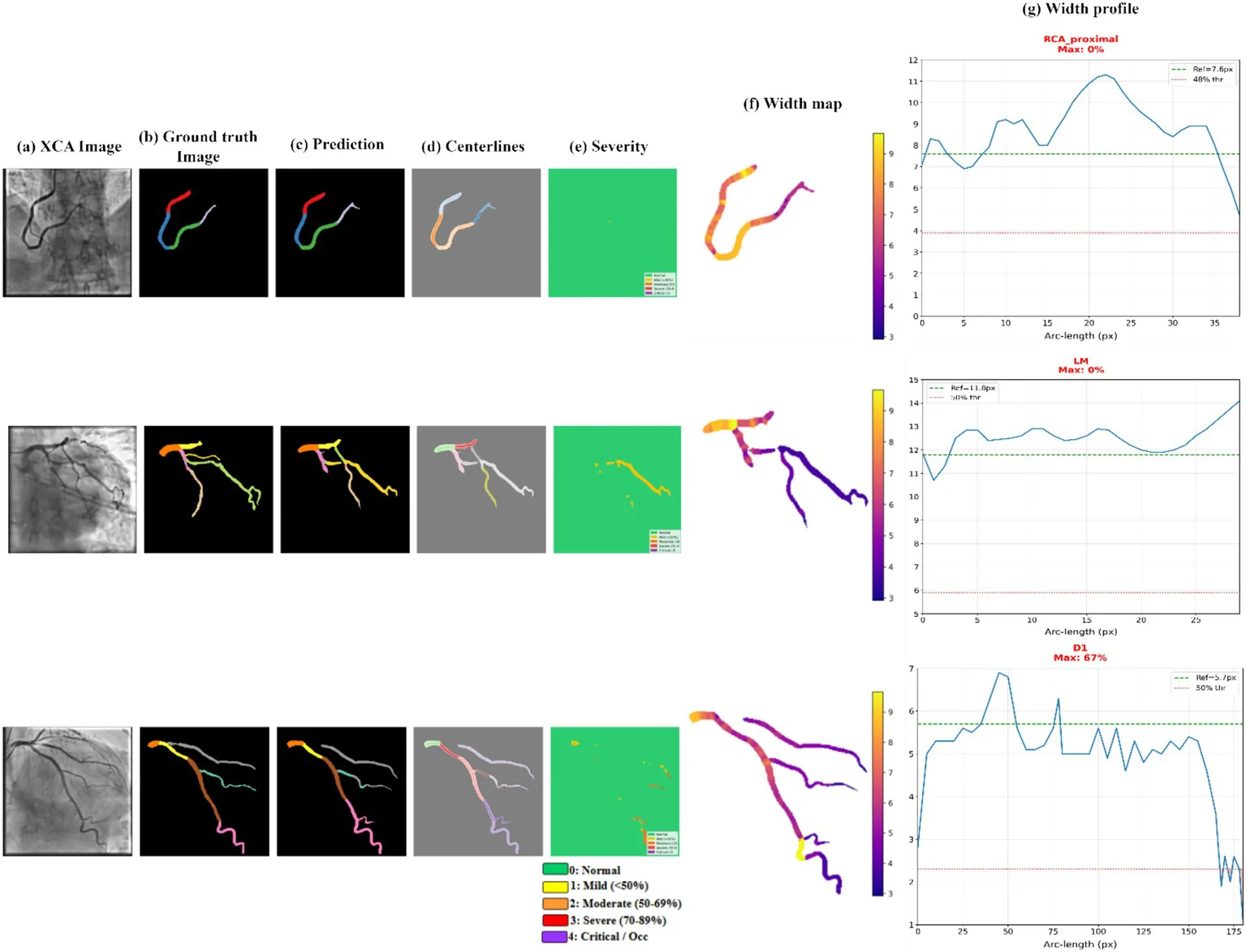
Qualitative pipeline results showing segmentation, stenosis severity, and SYNTAX score estimation on XCA images: **(a)** XCA image: input angiography frame, **(b)** Ground truth: Reference segmentation, **(c)** Prediction: predicted segmentation mask, **(d)** centrelines: extracted vessel centrelines, **(e)** severity: stenosis severity classification map, **(f)** width map: local vessel diameter along the centreline, **(g)** width profile: diameter (px) vs. arc-length (px), with reference and threshold lines.

**Figure 14:**
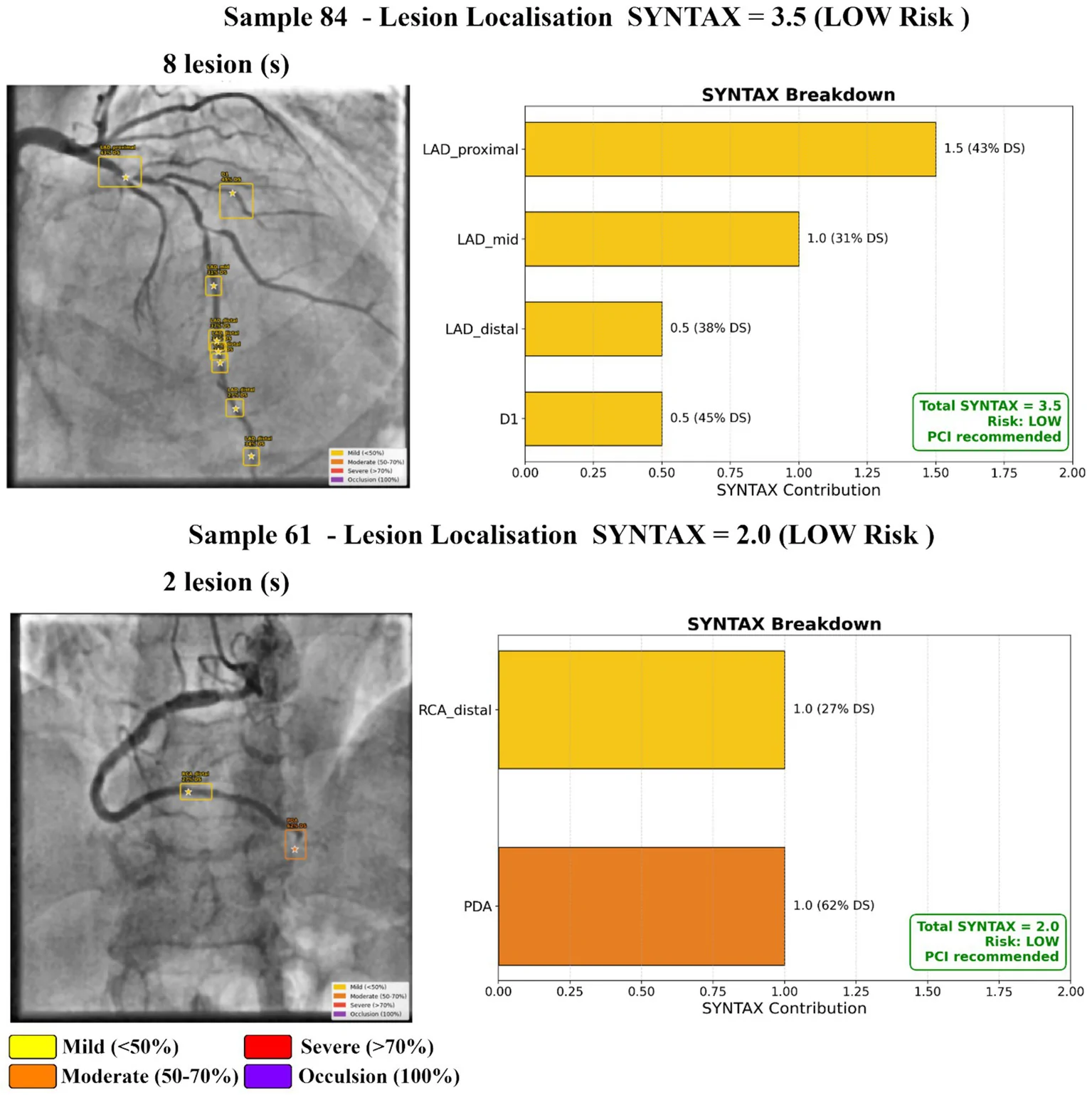
Coronary lesion localisation and risk-stratification via per-vessel SYNTAX score contribution.

**Figure 15:**
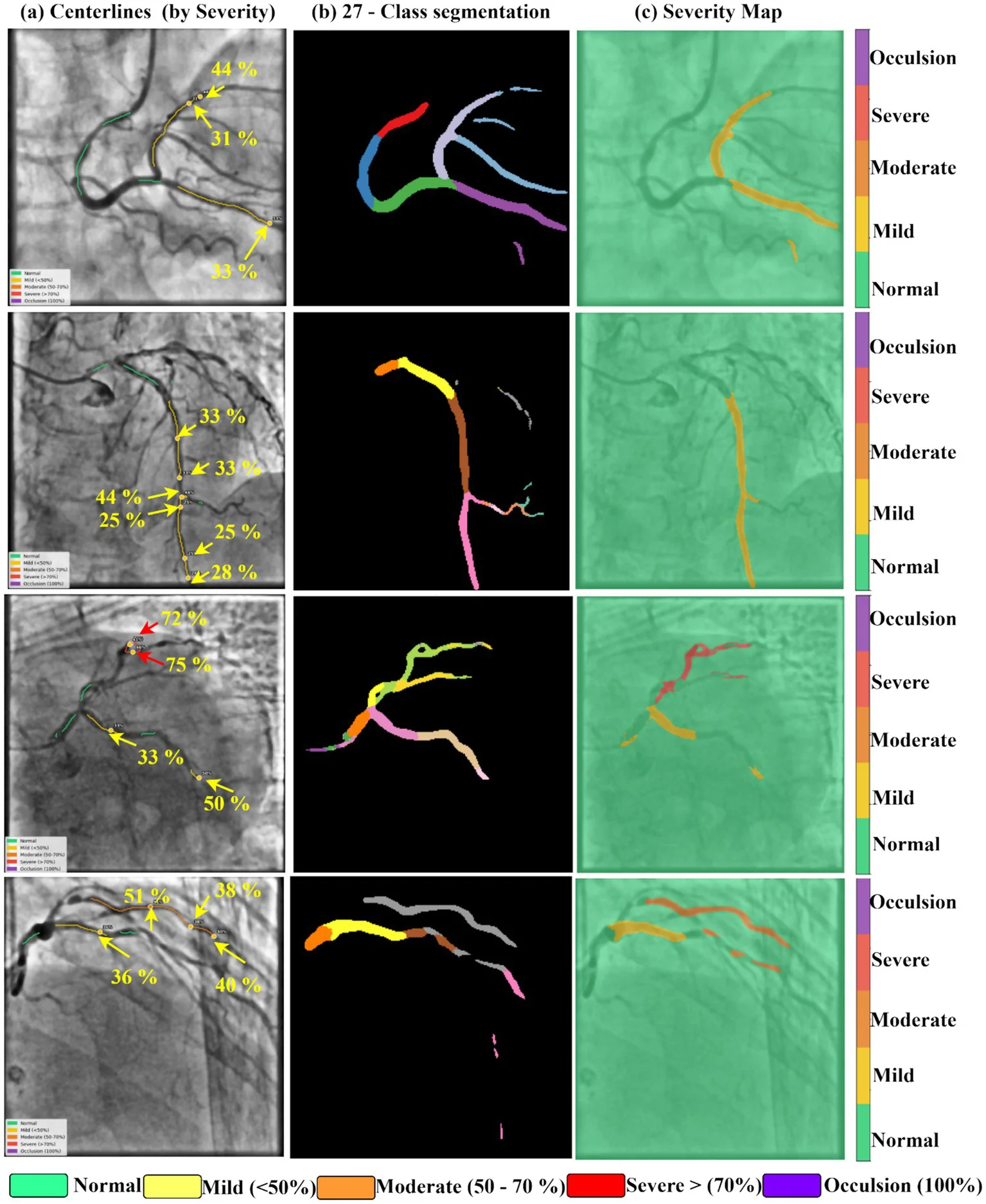
Coronary artery stenosis severity grading and segmentation overlay on XCA images **(a)** Extracted centrelines with detected stenotic regions and percentage diameter stenosis values. **(b)** Twenty-seven-class coronary artery segmentation showing anatomically labelled vessel branches **(c)** Severity map overlaid on the original XCA image.

#### 5.5.1 Centreline and ray casting

The Figure 11 shows the centreline extraction and perpendicular ray casting results on the segmented coronary vessels. Each vessel segment is converted into a single-pixel centreline, and perpendicular rays are cast along its length to measure the local vessel diameter. Regions with a noticeable reduction in diameter are identified as potential stenosis locations and highlighted in red. This stage provides accurate vessel diameter measurements and stenosis locations for quantitative severity evaluation in Stage 4.

The Figure 12 shows the severity of the stenosis map and vessel width profile for representative XCA images. Vessel regions are colour-coded according to the degree of narrowing, while the width profile compares the measured vessel diameter with the reference diameter and stenosis threshold. Areas where the vessel diameter falls below the threshold are identified as stenotic lesions. This analysis provides a quantitative measure of stenosis severity for each vessel segment and supports SYNTAX score calculation in Stage 5.

### 5.6 SYNTAX score estimation

The total SYNTAX score was calculated by adding the scores of all diseased vessel segments detected within an individual angiographic frame. Each segment score was obtained using the SYNTAX anatomical segment weight and the stenosis severity multiplier, adhering to the Sianos et al. (2005) scheme: a multiplier of 2 for non-occlusive lesions with 50–99% diameter stenosis, and 5 for total occlusions. Proximal segments such as Left Main, proximal RCA, and proximal LAD contributed more because of their higher clinical importance. The estimated SYNTAX scores for all test patients are shown in Table 10.

**Table 10:** Image-level SYNTAX score statistics.

Statistic	Value
Mean ±SD	7.1 ± 5.1
Median	5.5 (0.0–10.5)
Minimum	0.0
Maximum	21.5
Images with no detected lesion (*n*, %)	114 (38.0%)

Since the ARCADE dataset does not provide ground truth SYNTAX score labels, direct numerical validation was not feasible. The estimated scores are therefore supported by two forms of indirect evidence, First, population-level distribution, the estimated mean of 7.1 ± 5.1 is lower than the published clinical patient-level SYNTAX trial baseline of 14.5 ± 11.2. Second, clinical face validity, proximal segments consistently contributed higher scores than distal segments, in accordance with established SYNTAX methodology (Sianos et al., 2005). Neither constitutes lesion-level validation, and the SYNTAX scores reported here are presented as characterized pipeline outputs rather than as clinically validated measurements. All 300 test images were classified as low risk (maximum Score of 21.5) the intermediate and high-risk categories were not represented and require on multi-view angiographic data.

The Figure 13 shows the complete workflow of the proposed pipeline for a representative XCA image, including the input image, ground truth mask, predicted segmentation, extracted centrelines, stenosis severity map, vessel width map, and estimated SYNTAX score. The strong agreement between the ground truth and predicted segmentation demonstrates the accuracy of the earlier stages. The final SYNTAX score is calculated from the detected stenosis severity across vessel segments and is used to determine the corresponding risk category. These results highlight the effectiveness of the proposed pipeline in automatically estimating SYNTAX scores from XCA images.

Representative XCA images showing automatically detected stenotic lesions (boundary boxes). The corresponding bar charts present the contribution of each coronary artery segment to the total SYNTAX score based on the estimated percentage diameter stenosis, enabling patient risk classification and PCI recommendation.

The Figure 14 illustrates the detected coronary lesions and the SYNTAX score contribution of each affected vessel segment. Lesions are highlighted on the XCA image according to their severity, while the score breakdown identifies the vessel segments contributing to the total SYNTAX score. The estimated SYNTAX score and recommended treatment strategy are also displayed, providing a clear and interpretable evaluation of cardiovascular risk.

The Figure 15 shows stenosis severity grades overlaid on the predicted coronary artery segmentation and the original XCA image. Vessel segments are colour-coded based on the degree of narrowing, providing a clear visualization of coronary artery disease severity. The results demonstrate that the proposed pipeline can produce per-segment severity grades across multiple vessel segments within a single automated pipeline.

Standard mean Dice evaluates all 26 coronary vessel classes, including absent vessels, which can unfairly lower performance. Therefore, Present -class Dice was used to evaluate only vessel classes present in the image. The proposed method attained 0.8214 present class dice compared to 0.7512 standard mean Dice, showing improved and more accurate evaluation on the ARCADE Dataset.

In stenosis detection percent diameter stenosis was calculated by comparing vessel width at each centreline point with the proximal reference width. Per-class stenosis thresholds were optimised using the Aquila optimiser with higher priority given to reducing false negatives. The stenosis severity distribution for all vessel segments in the test set is shown in Table 9.

Only lesions with at least 50% diameter narrowing, located within the defined coronary segments which are ≥1.5 mm in calibre, were included in the SYNTAX score calculation, following the SYNTAX score methodology (Sianos et al., 2005).

Table 8 reports the Stage 3 outcome across the 300 test images centreline extraction yield, geometric measurements, lesion counts, and runtime. Measurement stability is quantified by rerunning the identical pipeline on ground-truth masks, isolating the effect of segmentation differences on Stage 3 geometric measurements. The mean absolute difference in percentage diameter reduction (|Δ%DS|) between predicted-mask and ground-truth mask measurements is 2.8% ± 1.4% for proximal and 6.5% ± 3.1% for distal branch segments as listed in Table 8. The larger distal error reflects the greater sensitivity of smaller-calibre vessels of (mean reference diameter 3.8 ± 0.9 px distal versus 9.6 ± 1.8 px proximal). Despite this, the severity-grade agreement between the two remains 95.14%, indicating that the boundary differences do not change the resulting stenosis grade in the large majority of segments.

## 6 Discussion

Our proposed work presents a five-stage pipeline for CAD evaluation from XCA images, designed to replicate the full clinical SYNTAX scoring workflow in a structured and interpretable approach. Each stage is motivated by a specific clinical challenge, and the sequential design ensures that clean information passes from one stage to the next stage. The first major challenge in XCA image analysis is the severe class imbalance between coronary vessel and pixels in background. Vessel pixels occupy less than 7% of the image, making it difficult for a basic multiclass segmentation model to learn vessel features effectively. To address this issue, Stage 1 performs binary vessel segmentation to separate vessel pixels from the background and generate a clear vessel mask before further analysis.

The segmentation network uses an EfficientNetV2-S encoder performed extraction of features efficiently. SS2D blocks and Linear projection improves feature learning between encoder and decoder, while QAG components help concentrates on vessel regions and suppress background interference. AGDrop regularization reduces overfitting, and SBHO automatically optimises loss weights and augmentation settings. The outcome is a precise binary vessel mask that serves as the foundation for all subsequent stages.

Stage 2 uses the binary vessel mask from Stage 1 to classify vessel pixels into 27 coronary artery segments. Since the vessels have already been separated from the background, the network can focus on detecting anatomical similar segments such as the LAD, LCX, RCA and their branches. A 4-channel guided input, consisting of the original XCA image and vessel guidance information, provides anatomical context. SS2D and QAG modules enhance feature learning and attention to vessel regions, while prototype learning improves the distinction between similar vessel segments. Test-time augmentation is used during inference to improve prediction stability. The output is a labelled 27-class coronary artery segmentation map that serves as the basis for subsequent geometric and stenosis analysis.

Stage 3 converts 27-class segmentation map into a geometric vessel representation. Each vessel segment is skeletonized to extract a centreline, and perpendicular rays are cast along the centreline to measure vessel width and generate a width profile. Regions where the width is significantly reduced compared to the reference diameter are detected as potential stenotic regions. The Aquila optimiser automatically refines the minimum-lumen position on extracted width profile, reducing sensitivity to single-pixel noise in the measured geometry. The output of Stage 3 consists of stenosis candidate locations and vessel width measurements, which are passed to Stage 4 for severity evaluation.

Stage 4 measures the severity of each stenosis detected in Stage 3. The percentage diameter stenosis is calculated by comparing the narrowed vessel width with the reference vessel diameter. Based on this value, each lesion is classified as mild, moderate, severe, or occlusion according to clinical scoring guidelines. This provides an objective and consistent evaluation of stenosis severity, reducing variability in manual interpretation.

Stage 5 combines the stenosis severity grades from stage 4 to calculate the final SYNTAX score. Each diseased vessel segment contributes a weighted score based on its anatomical importance and lesion severity. The total score is used to classify patients into low, intermediate, or high-risk categories and to support treatment decisions such as PCI or CABG. A per-vessel score breakdown is also provided to detect the segments contributing most to the overall risk. The proposed five-stage pipeline divides SYNTAX score estimation into clear and manageable tasks, vessel segmentation, vessel classification, stenosis detection, severity grading, and score calculation. This design improves accuracy, maintains clinical interpretability, and allows each stage to be evaluated independently. By integrating EfficientNetV2S, SS2D, QAG, prototype learning, test-time augmentation, the aquila optimiser, and SBHO, the pipeline delivers efficient and SYNTAX Score estimation from XCA images.

The limitation of this work is that the ARCADE dataset does not include patient-specific ground-truth SYNTAX scores. Consequently, the estimated scores could not be directly compared with cardiologist derived reference scores. Further validation on datasets with clinical-annotated SYNTAX scores is required to confirm the reliability of the estimated scores.

## 7 Conclusion

We proposed a five-stage automated pipeline for evaluating coronary artery disease from XCA images and estimating the SYNTAX score. The pipeline performs binary vessel segmentation, 27- class coronary artery segment labelling, centreline extraction and stenosis detection, stenosis severity grading, and SYNTAX score calculation, providing an end-to-end framework for coronary artery analysis. Each stage was designed to solve a specific challenge in the pipeline. Stage 1 handles class imbalance problem and generates a binary vessel mask using EfficientNetV2-S, SS2D, QAG, AGDrop, and SBHO. Stage 2 classifies the vessel pixels into 27 coronary artery segments using guided input, prototype learning, and test-time augmentation. Stage 3 extracts vessel centrelines, measures vessel width, and detects possible stenotic regions with the help of Aquila optimiser. Stage 4 determines the severity of each stenosis as mild, moderate, severe or occlusion. Finally, Stage 5 combines the severity information to calculate the SYNTAX score and support treatment decisions. Our proposed VMamba-QAG-Net was evaluated on the ARCADE dataset and performed effectively in both segmentation tasks. For binary vessel segmentation, the model attained a Dice coefficient of 0.9070, Jaccard Index of 0.8294, accuracy of 0.9841, precision of 0.8950, recall of 0.9210, and specificity of 0.9921. For 27-class coronary artery segmentation, attained a macro-Dice coefficient of 0.7512, a present class Dice of 0.8214, a Jaccard Index of 0.6018, accuracy of 0.9514, precision of 0.7248, recall of 0.7134, specificity of 0.9763. These results show the effectiveness of the proposed framework for accurate vessel and coronary artery segment analysis and clinical decision support tool for SYNTAX score estimation. Future work will focus on clinical validation and extension to multi-view angiographic images.

## Data Availability

Publicly available datasets were analyzed in this study. This data can be found here: X-ray coronary angiograms ARCADE dataset is publicly available in Zenodo. https://zenodo.org/records/10390295; https://data.mendeley.com/datasets/9fb9b4fngf/1.
